# Demonstration of doxorubicin's cardiotoxicity and screening using a 3D bioprinted spheroidal droplet-based system†‡

**DOI:** 10.1039/d3ra00421j

**Published:** 2023-03-13

**Authors:** Raven El Khoury, Salma P. Ramirez, Carla D. Loyola, Binata Joddar

**Affiliations:** a Inspired Materials & Stem-Cell Based Tissue Engineering Laboratory (IMSTEL), The University of Texas at El Paso El Paso TX 79968 USA bjoddar@utep.edu; b Department of Metallurgical, Materials, and Biomedical Engineering, M201 Engineering, The University of Texas at El Paso 500 W. University Avenue El Paso TX 79968 USA; c Border Biomedical Research Center, The University of Texas at El Paso 500 W. University Avenue El Paso TX 79968 USA

## Abstract

Doxorubicin (DOX) is a highly effective anthracycline chemotherapy agent effective in treating a broad range of life-threatening malignancies but it causes cardiotoxicity in many subjects. While the mechanism of its cardiotoxic effects remains elusive, DOX-related cardiotoxicity can lead to heart failure in patients. In this study, we investigated the effects of DOX-induced cardiotoxicity on human cardiomyocytes (CMs) using a three-dimensional (3D) bioprinted cardiac spheroidal droplet based-system in comparison with the traditional two-dimensional cell (2D) culture model. The effects of DOX were alleviated with the addition of *N*-acetylcysteine (NAC) and Tiron. Caspase-3 activity was quantified, and reactive oxygen species (ROS) production was measured using dihydroethidium (DHE) staining. Application of varying concentrations of DOX (0.4 μM–1 μM) to CMs revealed a dose-specific response, with 1 μM concentration imposing maximum cytotoxicity and 0.22 ± 0.11% of viable cells in 3D samples *versus* 1.02 ± 0.28% viable cells in 2D cultures, after 5 days of culture. Moreover, a flow cytometric analysis study was conducted to study CMs proliferation in the presence of DOX and antioxidants. Our data support the use of a 3D bioprinted cardiac spheroidal droplet as a robust and high-throughput screening model for drug toxicity. In the future, this 3D spheroidal droplet model can be adopted as a human-derived tissue-engineered equivalent to address challenges in other various aspects of biomedical pre-clinical research.

## Abstract 2

A unique 3D bioprinted cardiac spheroidal droplet model used to test the cytotoxic effects of DOX.

## Graphical abstract

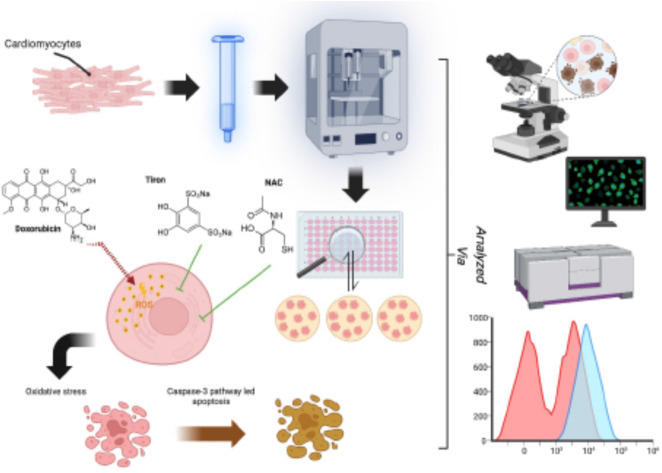

## Introduction

We adopted a 3D cardiac spheroidal droplet model in this study to test the toxic effects of doxorubicin on cardiomyocytes (CMs)^1,2^ to overcome challenges associated with conventional modes of drug delivery. In preclinical studies, researchers have used 2D CM cultures as a prevalent method to assess drug response and cardiotoxicity.^3^ However, 2D cultured CM models lack cell–cell and cell–matrix interactions and fail to mimic the *in vivo* microenvironment of the native heart. This increases the need for *in vitro* 3D cardiac tissue models for more effective drug toxicity testing and pharmaceutical assays.^1^ 3D bioprinting is an additive manufacturing process involving biomaterials, living cells, and active biomolecules aiming to fabricate structures that mimic natural tissue characteristics and an extracellular matrix (ECM) environment capable of sustaining cell adhesion, proliferation, and differentiation.^4^ Many studies have used biomaterials such as alginate, gelatin, collagen, fibrinogen, and hyaluronic acid. CMs seeded in such hydrogel-based scaffolds retain cardiac, and other cell specific-functions as these scaffold-based systems can provide an ideal 3D culture environment for CMs and other cardiac cells.^5–14^

In a recently published study, a unique droplet-based extrusion printing approach was performed to 3D bioprint cardiac spheroids using a CELLINK-BIO X printer. This technique was further scaled up to a high throughput 96-well array set-up with a six-axis robotic arm using a 3D bioprinter (BioAssemblyBot). This study produced morphologically consistent 3D spheroidal droplets with significant porosity and a large degree of pore interconnectivity. Moreover, the scaffolds retained structural fidelity after 28 days confirming their use in long-term *in vitro* cell culture studies. Rheological studies performed on these 3D spheroidal droplets were found to emulate Young's modulus of the native cardiac tissue making this an attractive model for *in vitro* studies. Cell viability quantification showed a steady turnover of cells in the scaffolds for up to 14 days, and the percent (%) heterocellular coupling (HC) between CMs and cardiac fibroblasts (CFs) was ∼80% in the 3D spheroidal droplets. This led to the fabrication of a high-throughput 3D cardiac tissue model that can be applied for studying drug effects on cardiac cells.^1^ For this study, we hypothesized that by using such a 3D bioprinted cardiac spheroidal model, we would be able to build a robust high-throughput 3D model for drug toxicity testing.

The anthracycline drug, doxorubicin (DOX), is one of the most potent antineoplastic agents used to treat various malignancies, including lymphoma, leukemia, and other solid tumors.^15,16^ Its use has been restricted due to its cardiotoxic effects, especially in patients at different stages of heart failure. Several hypotheses have been proposed regarding the mechanisms of DOX-induced cardiomyopathy.^17^ The generation of ROS is one route by which DOX harms the myocardium. Furthermore, free radical oxygen and lipid peroxidation play other essential roles in the pathogenesis of DOX-induced cardiomyopathy. In addition, it has been reported that apoptosis plays a significant role in the development of heart failure in humans by inducing autophagy in cardiomyocytes.^3,15,16,18^

Based on such an existing premise, we adopted a 3D bioprinted cardiac spheroidal model for testing the effects of cardiotoxicity induced by DOX.^1^ To do this, CMs were 3D bioprinted in a high throughput fashion inside of all wells in a 96-well plate. After confirmation of cardiac toxicity with DOX, quantitatively *via* a tetrazolium salt assay (MTS) and qualitatively by conducting a live/dead assay, we then aimed to mitigate these cytotoxic effects using two well-known antioxidants, *N*-acetyl cysteine (NAC) and Tiron. Furthermore, we quantitatively assessed the proliferation trends of CMs using a flow cytometer, analysed the activation of the caspase-3 pathway, and the release of ROS in the presence of DOX and the antioxidants (Tiron and NAC).

This study yielded 3D spheroidal droplet scaffolds specifically tailored to study the induced cytotoxic effects of DOX *in vitro*. We anticipate the use of this 3D bioprinted cardiac cell model to facilitate early-phase drug development in preclinical studies with sufficient versatility to evaluate the responses of various drugs and small molecules more efficiently at a relatively low cost and in a high throughput manner.

## Experimental

### Reagents and chemicals

Gelatin type A (MP Biomedicals LLC, USA, Cat. no. 901771) and medium viscosity alginic acid (MP Biomedicals LLC, USA, Cat. no. 154724) were used to fabricate the hydrogel scaffolds used in this study. Calcium chloride crosslinking solution was produced from calcium chloride dihydrate (Fisher Chemical, Germany, and CAS. no. 10035-04-8) and phosphate-buffered saline (PBS) 10× solution (Thermo Fisher Scientific, USA, Cat. no. 70011069). AC16 human cardio-myocytes (ATCC, Manassas, VA) were cultured and expanded in Dulbecco's modified Eagle medium (Sigma, Germany, Cat. no. D6434) containing 2 mM l-glutamine (EMD Millipore, Germany, Cat. no. TMS-002-C), 12.5% FBS (EMD Millipore, Germany, Cat. no. ES-009-B) and 1× penicillin–streptomycin solution (EMD Millipore, Germany, Cat. No. TMS-AB2-C), 0.25% trypsin-EDTA (Thermo Fisher Scientific, USA Cat. no. 25200056). 96 round-bottom well plates (Thermo Fisher Scientific, USA, Cat. no. 12-565-212) were used for bioprinting and cell culture. Doxorubicin hydrochloride powder (Sigma, Germany, CAS-no: 25316-40-9) was used as a cardiotoxic agent. *N*-Acetyl-cysteine (Sigma, Germany, CAS-no: 616-91) was obtained from Sigma-Aldrich and Tiron (Thermo Fisher Scientific, USA, Cat. No. 174140250) were used as ROS scavenging agents. MTS Assay Kit (Colorimetric) (ab197010) was procured from Abcam, MA, USA and the CellTiter 96® Aqueous One Solution Cell Proliferation Assay (Promega, USA, Cat. no. G3582) was used to determine cell viability. Hanks' Balanced Salt Solution (HBSS) (Thermo Fisher Scientific, USA, Cat. no. 88284) was used for culture washes. The LIVE/DEAD® Viability/Cytotoxicity Kit (Thermo Fisher, USA, Cat. no. L3224) was used to image viable and dead cells. The caspase-3 Colorimetric Assay Kit (NucView® 488 Caspase-3 Assay Kit for Live Cells, USA, Cat. no. 30029-T) was purchased from Biotium (USA) to detect cellular apoptosis. CellTrace Violet, proliferation dye (Invitrogen, USA, Cat. no. C34557) was used for tracking cell proliferation, and dihydroethidium (DHE) was used as a superoxide indicator (Thermo Fisher Scientific, USA, and Cat. no. D11347).

### DOX and Tiron/NAC solution preparation

10 g of DOX was dissolved in 1.72 mL of DMSO to reconstitute a stock solution of 10 mM according to the manufacturer's protocol.^19,20^ To induce cardiotoxicity in the 3D spheroidal droplets with CMs, four different stock concentrations of DOX (40 μM, 60 μM, 80 μM, and 100 μM) were prepared and 2 μL of each stock solution was added to 200 μL of culture media. DOX induces the production of ROS and antioxidants, such as NAC and Tiron can mitigate ROS-related cytotoxic effects.^21,22^ From a 200 mM Tiron/NAC stock solution, 1 μL and 3 μL were added to 200 μL of culture media to prepare a solution of 1 mM and 3 mM respectively. Similarly, from a 500 mM Tiron/NAC stock solution, 2 μL and 3.2 μL were added to 200 μL culture media to form a solution of 5 mM and 8 mM respectively and from a 613 mM stock solution, 3.3 μL and 4.9 μL were added to form a solution of 10 mM and 15 mM respectively. To ensure their homogeneous diffusion into the 3D bioprinted scaffolds and in the 2D samples, the agents were added 24 h prior to the addition of DOX.^23–25^ MTS measurements were recorded on a microplate reader (BioTek Synergy H1, CA, USA) on days 1, 3, and 5. The blank samples included the 3D scaffolds only (no cells) with DOX. For a more effective comparison, both positive (in the presence of DOX) and negative (in the absence of DOX) 2D control samples were included.

### Cell culture

AC16 human CMs (passages 3–4) were cultured in Dulbecco's modified Eagle's complete growth medium supplemented with 10% fetal bovine serum (FBS) and 1% penicillin–streptomycin. Before 3D bioprinting, cells were harvested by trypsinization and mixed with the alginate–gelatin hydrogel to constitute a final cell seeding density of 1 × 10^6^ cells per 1 mL of bioink (approximately 50 spheroidal droplets/1 mL bioink; 20 000 cells per spheroidal droplet). Cultures were incubated with a complete growth medium and maintained in a humidified atmosphere of 95% air and 5% CO_2_ at 37 °C. The initial cell seeding density used in this study was 20 000 cells per 2D well and 20 000 per 3D spheroidal droplet.^1^

### Bioink preparation

An optimized protocol for bioink preparation was based on an alginate/gelatin scaffold that was developed and reported in a previous published study from our group.^1^ Briefly, under aseptic conditions, 2% w/v gelatin and 3% w/v alginate were dissolved in Milli-Q water respectively under constant stirring.^1,5,26^ The mixture was next allowed to rest and dissolve for 16–24 h at room temperature and centrifuged at 1200 rpm for 5 min to remove the remaining air bubbles. Before cell printing, gels were additionally UV-sterilized for 15 min, after which they were loaded into a 3 mL syringe (CELLINK, Blacksburg, VA, USA).

### Biofabrication of 3D constructs and culture

A 3D spheroid with a diameter of 2 mm was designed using SolidWorks® software. Using CELLINK BIO X (Blacksburg, VA, USA), the temperature-controlled printer head was used to place the droplets inside a 96-round-bottom well plate. Printing parameters are shown in Table 1, below. 5 μL of 80 mM CaCl_2_ sterile solution was added to the bottoms of each of the wells. The resultant spheroidal droplets were further cross-linked with an additional 75 μL of CaCl_2_ post-printing while being placed on a Belly Dancer Shaker (IBI SCIENTIFIC, Iowa, USA) for 15 min at a speed of 4.5 (au).

**Table 1:** Parameters used for 3D bioprinting of spheroidal droplets

Printer setting	Requirement
Printing pressure	13–15 kPa
Printing speed	0.7 mm s^−1^
Nozzle size	16G (∼1.9 mm)
Temperature	Room temperature (25–28 °C)

### MTS standard curve for cardiomyocytes

CM cell viability was determined using CellTiter 96 Aqueous One Solution Cell Proliferation Assay kit from Promega (Madison, WI). The culture medium was removed, and the MTS tetrazolium salt was prepared and added in the ratio of 1 : 10 (MTS solution : media) where the samples were left in the incubator (5% CO_2_ and 37 °C) for 4 hours according to the manufacturer's protocol.^5,27,28^ Absorbance was recorded on a microplate reader (BioTek Synergy H1, CA, USA) at 490 nm. Using the calibration curve, the number of live cells was determined, and the percentage of surviving cells was compared with that of the control sample from the equations shown below:

The linear best-fit equation for 3D spheroidal droplets used was:

1 *y* = 3.1 × 10^−5^*x* + 0.035

The linear best-fit equation for 2D samples used was:

2 *y* = 3.6 × 10^−5^*x* + 0.074

3
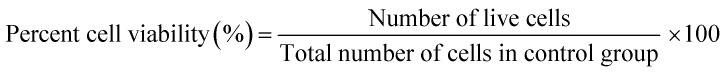


To plot a standard curve, varying concentrations of CMs were used to determine the MTS value for each concentration and construct a best fit calibration curve for both 3D bioprinted spheroidal cell droplets and 2D cell culture samples. This enabled us to quantify the number of live cells *via* its corresponding linear equation derived using MATLAB by entering the variable “*y*” as the OD value and calculating “*x*” as the number of viable cells for both 3D and 2D samples using the above two eqn (1) and (2)^28,29^ and percent cell viability (% CV) was deduced using eqn (3).

### Live/dead assay

Live/dead cytotoxicity assay assessed cell survival following the manufacturer's protocol. NAC and Tiron were added to the 3D and 2D samples, 24 h prior to the addition of DOX. Based on the intracellular esterase activity and plasma membrane integrity, calcein AM was used to stain live cells in green exclusively. In contrast, the ethidium homodimer dye was used to stain only the compromised plasma membranes of dead cells by binding to nucleic acids exhibiting a red fluorescence dye. Images were acquired with an inverted Zeiss microscope (Zeiss, AXIO, Germany) using the filter set, 43 DsRed (ex533–558 nm/em570–640 nm) to observe dead cells and 38 green fluorescent Prot (ex450–490 nm/em500–550 nm) to observe live cells. Percent cell viability was quantified using the eqn (4) below:4
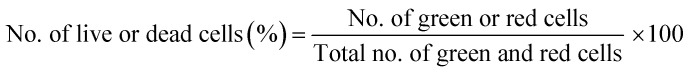


### *In vitro* caspase-3 activity assay

Activation of the caspase-3 (Cas-3) pathway is considered a pivotal event during cell apoptosis; therefore, Cas-3 activity was determined using NucView® 488 Cas-3 substrate; a permeable fluorogenic caspase substrate for identifying Cas-3 upregulation within live cells.^30,31^ The substrate comprises of a fluorogenic DNA dye coupled with a DEVD (Asp–Glu–Val–Asp) substrate element specific for caspase-3 and was prepared according to the manufacturer's protocol. The DEVD/Cas-3 recognition subunit is non-fluorescent until cleaved. During apoptosis, the substrate enters the cytoplasm by crossing the cell membrane, where it is cleaved by Cas-3. The dye, NucView®488, enters the cell nucleus where it binds with DNA and fluoresces green at 488 nm, expressing apoptosis. 15 mM of Tiron/NAC was added to the 3D and 2D samples 24 h prior to the addition of DOX, and cas-3 activity was quantified using a microplate reader (BioTek Synergy H1, CA, USA) on days 1 and 3 and high magnification images were acquired using an LSM 700 confocal microscope (ZEISS LSM, Germany).

### Dihydroethidium (DHE) staining

To determine the level of ROS production in DOX-induced AC16 CMs, intracellular oxidant production levels in CMs were measured using DHE fluorescence following the manufacturer's protocol. NAC and Tiron were added to the 3D and 2D samples 24 h prior to the addition of DOX, and at each time point samples were washed with Hanks' balanced salt solution (HBSS) and incubated with DHE for 30 min at 37 °C. The cells were washed 3 times, and mean fluorescent intensity readings were taken using a microplate reader (BioTek Synergy H1, CA, USA) on days 1 and 3. Images were taken using an inverted Zeiss microscope (Zeiss, AXIO, Germany) using the filters, 43 DsRed (em533–558 nm/ex570–640 nm) to observe ROS and 49 DAPI (ex335–383 nm/em420–470 nm) as an overall nuclear stain.

### Assessment of cell viability with DOX and NAC using flow cytometry

Flow cytometric analysis was performed using Beckman Coulter Gallios Flow Cytometer (Beckman Coulter, CA, USA). CMs were pre-stained using Cell Trace Violet (CTV) proliferation kit (Invitrogen, CA, USA) according to the manufacturer's protocol and were treated with their respective doses of NAC and DOX, as described earlier. On day 1 and day 3, the 3D spheroidal droplets with cells were cut using a blade, and cells were extracted using Miltenyi gentleMACS Dissociator (Miltenyi Biotec, MA, USA) using a Multi tissue Dissociation Kit-1 by running the Multi_B program according to the manufacturer's protocol. For 2D samples, cells were detached using trypsin-EDTA. Cells were fixed with 4% paraformaldehyde (PFA) for 15 min at room temperature, then added to their assigned FACS analysis falcon tubes, and analysed using excitation and emission wavelengths of 405 and 450 nm, respectively. Negative controls included freshly isolated non-stained cells and positive controls were pre-stained with CTV.^1,5,32^

### Quantitative reverse transcriptase chain reaction (qPCR) analysis

To compare the gene expression and integrity between the CMs present in the 3D bioprinted spheroidal hydrogels to CMs cultured on 2D surfaces, qPCR was performed. After 5 days of culture, scaffolds were washed using 1× PBS, and cells were extracted following previously published methods.^33^ The extracted mixture was then centrifuged at 400 g for 10 min yielding the cell pellet.

Total RNA extraction was carried out using RNeasy® Plus Mini Kit (QIGEN, Germany) according to the manufacturer's instructions. Extracted RNAs were quantified by NanoDrop OneC spectrophotometer (ThermoFisher Scientific, MA, USA), and the absorbance ratios at 260/280 nm and 260/230 nm were measured to control RNA purity as shown in Table 2, below.

**Table 2:** Quantification and absorbance of total RNA

Sample cardiomyocytes	ng μL^−1^	260/280	230/260
Control 25 mM Glu	16.7	2.15	0.15
Cells in 3D structure no. 1	12.4	1.95	0.48
Cells in 3D structure no. 2	6.5	1.85	0.14
Cells in 3D structure no. 3	10	1.70	0.11

Total RNA (50 ng) was reverse transcribed to cDNA using the First Strand cDNA Synthesis Kit (OriGene Technologies Inc., MD, USA) in a volume of 20 μL, according to the manufacturer's instructions. Extracted cDNA was quantified by NanoDrop OneC spectrophotometer (ThermoFisher Scientific, MA, USA), and the absorbance ratios at 260/280 nm and 260/230 nm were measured.

The RT-qPCR reactions were performed in Quantstudio 3 (ThermoFisher Scientific, MA, USA). The cardiomyocytes isolated from 2D and 3D samples (*n* = 3 each) were placed in qPCR tubes, with optical strip caps 3 (ThermoFisher Scientific, MA, USA) in a reaction volume of 20 μL. To avoid sample contamination and primer-dimer formation that could produce false positive results, no template control was used. The genes analysed were selected based on vendor's recommendations. The gene expression of GJA1 (Connexin 43) was studied as the target gene of interest and GAPDH was used as the reference gene (control); the access gene and the sequence primers are shown in the Table 3. The reaction started with a 10 min initial denaturation step at 95 °C, 40 cycles of 95 °C for 15 s and 60 °C for 15 s according to the protocol provided by Origene. The quantification cycle (CT) values were automatically calculated by the qPCR instrument software Quantstudio 3 (ThermoFisher Scientific, MA, USA). A statistical algorithm was used to evaluate quantitative gene expression using the comparative CT method (2-ΔΔCT).^34^ Average CTs of GAPDH was used as endogenous control and the stability of the target genes was expressed as Ct values of each candidate gene normalized with GAPDH.

**Table 3:** Sequence primers and access gene

Gene mix primer	Forward	Reverse	Access gene no.
GAPDH	GTCTCCTCTGACTTCAACAGCG	ACCACCCTGTTGCTGTAGCCAA	NM_002046
GJA1	GGAGATGAGCAGTCTGCCTTTC	TGAGCCAGGTACAAGAGTGTGG	NM_000165

## Results

### DOX affects the viability and proliferation of CMs in a dose-responsive manner

**Fig. 1:**
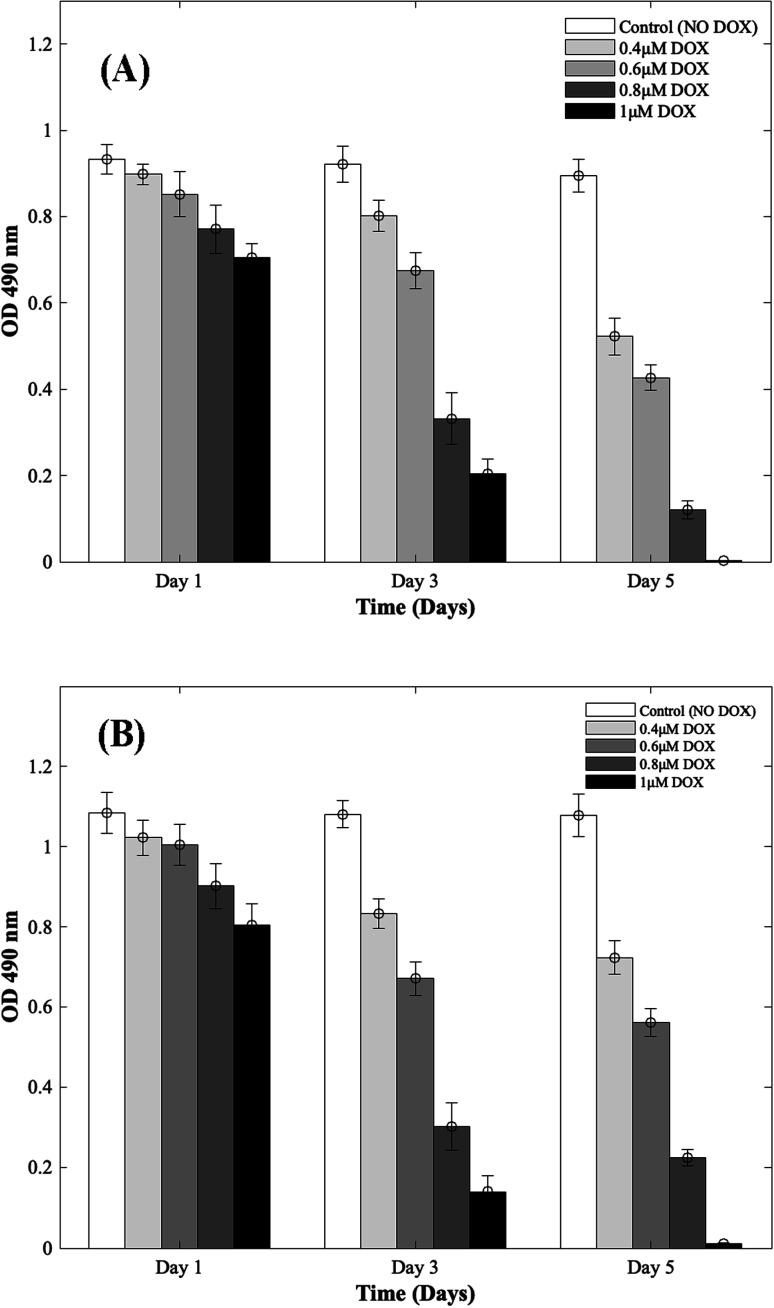
Dose responsive effects of DOX on CMs. Optical density measurements for MTS assay of CMs treated with increasing concentrations of DOX during 5 days of culture. (A) 3D bioprinted spheroidal droplets. (B) 2D samples. Optical density measurements for MTS assay of CMs treated with increasing concentrations of DOX during 5 days of culture. The actual cell numbers used to generate % CV are shown in Fig. S1-b and S8-b.†

Four different concentrations of DOX (0.4 μM, 0.6 μM, 0.8 μM, and 1 μM) were used to study the cardiotoxic effects on CMs using the 3D bioprinted spheroidal model, and the results were compared with 2D models. In Fig. 1 (for 3D and 2D results), MTS assay trends revealed that the OD values progressively reduced with respect to the control group (no DOX). Since the OD value is a measure of metabolic activity, it indicates the number of live cells per sample. Thus a lower OD value reflects a lower number of viable cells present in a sample and *vice versa*. For the 3D bioprinted spheroidal droplets, as shown in ESI, Table 1(A),† the OD value decreased from 0.90 ± 0.02 on day 1 to 0.52 ± 0.04 on day 5 (*p* = 0.001) when 0.4 μM of DOX was added, from 0.85 ± 0.05 on day 1 to 0.43 ± 0.03 on day 5 when 0.6 μM of DOX was added (*p* = 0.02) and from 0.77 ± 0.06 at day 1 to 0.12 ± 0.02 on day 5 when 0.8 μM of DOX was added (*p* = 0.005). With 1 μM of DOX, the OD value decreased from 0.71 ± 0.03 on day 1 to 0.002 ± 0.001 on day 5 (*p* = 0.015) but in the samples where DOX was not added, no statistical significance was observed between day 1 (0.93 ± 0.03) and day 5 (0.90 ± 0.04) values with *p* > 0.05. Results showed as the concentration of DOX was increased, the OD value was decreased correlating with a lesser number of viable cells confirming the cardiotoxic effects caused by DOX on CMs.

For the 2D CMs models, as shown in ESI, Table 1(B),† the OD value decreased from 1.02 ± 0.04 on day 1 to 0.72 ± 0.04 on day 5 (*p* = 0.035) when 0.4 μM of DOX was added, from 1 ± 0.05 on day 1 to 0.56 ± 0.04 on day 5 when 0.6 μM of DOX was added (*p* = 0.025) and from 0.90 ± 0.06 at day 1 to 0.22 ± 0.02 on day 5 when 0.8 μM of DOX was added (*p* = 0.005). With 1 μM of DOX, the OD value decreased from 0.81 ± 0.05 on day 1 to 0.011 ± 0.003 on day 5 (*p* = 0.03) but in the samples where DOX was not added, no statistical significance was observed between day 1 (1.08 ± 0.05) and day 5 (1.08 ± 0.05) values with *p* > 0.05. These trends correlated well with results from 3D samples.

To calculate the % cell viability (% CV) (eqn (1)) of CMs in each sample, the linear best-fit equations (eqn (2) and (3)) from the MTS standardization assay were used. For the 3D bioprinted spheroidal droplets (Fig. 1A/ESI, Table 1A†), the % CV decreased from 97 ± 2% on day 1 to 56 ± 4% on day 5 when 0.4 μM DOX was added, from 91 ± 5% on day 1 to 46 ± 3% at day 5 when 0.6 μM DOX was added, from 82 ± 6% at day 1 to 10 ± 2% at day 5 when 0.8 μM DOX was added and from 75 ± 3% at day 1 to 0.22 ± 0.11% at day 5 when 1 μM of DOX was added. These values confirmed the OD values from MTS assay reported earlier. All the trends were statistically significant between the varying time points studied.

For the 2D models (Fig. 1B/ESI, Table 1B†), % CV of CMs decreased from 94 ± 4% on day 1 to 64 ± 4% on day 5 when 0.4 μM DOX was added, from 92 ± 5% on day 1 to 48 ± 3% on day 5 when 0.6 μM DOX added, from 82 ± 6% at day 1 to 15 ± 1% at day 5 when 0.8 μM of DOX was added and from 73 ± 5% at day 1 to 1.02 ± 0.28% at day 5 when 1 μM DOX was added. The actual number of live CMs was derived from the best-fit curve using their corresponding OD values found in ESI, Fig. S1† (corresponding to 3D samples) and ESI, Fig. S2† (corresponding to 2D samples). Images showing the diffusion of DOX into the hydrogel scaffolds can be found in ESI, Fig. S3.† All the trends were statistically significant between the varying time points studied.

The effect of DOX on CMs was seemingly more pronounced in 3D scaffolds as confirmed by our results. This can be attributed to the intensified distribution of DOX inside the hydrogel's mesh network and its anchorage to the polymer's backbone that constitutes the bioink^35,36^ in contrast to 2D cell models where molecules can diffuse freely throughout the system.^37^

### Reversal of DOX-induced cardiotoxicity on CMs with the addition of NAC and Tiron

**Fig. 2:**
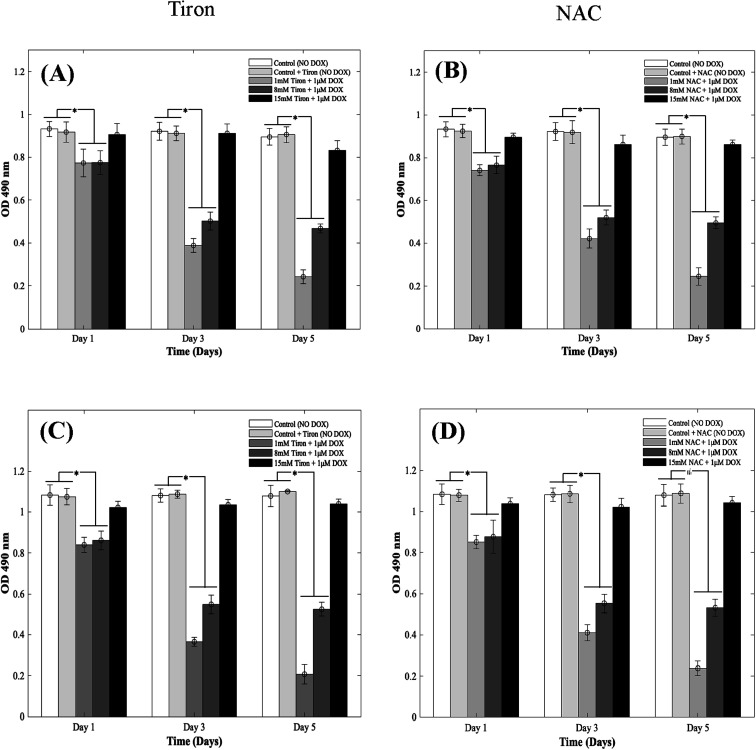
Quantitative analysis depicting the effects of supplementing Tiron/NAC on CMs. Optical density measurements for MTS assay of CMs treated with increasing concentrations (1 mm, 8 mm, and 15 mm) of (A) Tiron and (B) NAC with 1 μm DOX in using 3D spheroidal droplets and (C) Tiron and (D) NAC with 1 μm DOX in 2D samples.

**Fig. 3:**
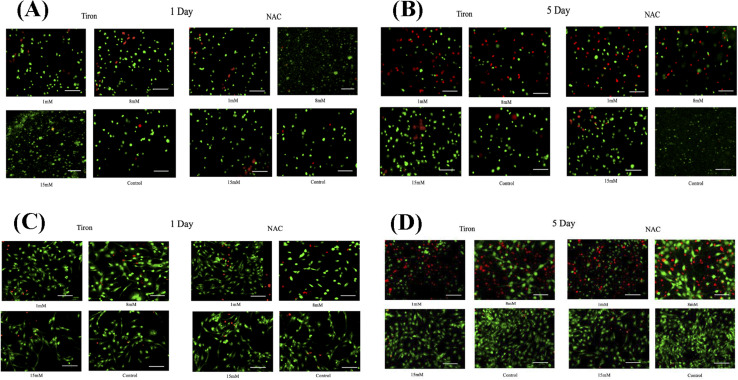
Live/dead assay analysis representing the effects of supplementing Tiron/NAC on cms using 3D spheroidal droplets. Representative fluorescence images of live/dead staining of cms treated with increasing concentrations (1 mm, 8 mm, and 15 mm) of Tiron and NAC respectively and 1 μm DOX. Live cells are stained in green by calcein AM and dead cells stained in red by ethidium homodimer after 1 day and 5 days of culture in 3D cultures (A and B) and 2D cultures (C and D). The scale bar corresponds to 100 μm.

DOX induces myocardial damage to the heart *via* the elevation of ROS.^38–40^ In an attempt to mitigate the cytotoxic effects caused by doxorubicin on CMs in the 3D spheroidal droplet model, six different concentrations of NAC and Tiron were initially tested. Preliminary MTS assay data for 3 mM, 5 mM, and 10 mM of antioxidants can be found in ESI, Fig. S4–S7† respectively. Quantitative analysis using a colorimetric assay with increasing concentrations of Tiron and NAC (Fig. 2A and B) in the presence of 1 μM DOX is depicted during 5 days of culture. For the 3D bioprinted spheroidal droplets, OD values (ESI, Tables 2A and B†) decreased from 0.77 ± 0.06 to 0.24 ± 0.03 (*p* = 0.002) and from 0.78 ± 0.06 to 0.47 ± 0.02 (*p* = 0.015) after 5 days of culture when 1 mM and 8 mM of Tiron were added respectively. But with the addition of 15 mM of Tiron, no statistically significant difference was observed between day 1 (0.91 ± 0.05) and day 5 (0.83 ± 0.05) (Fig. 3) with *p* > 0.05. With NAC, the OD value also dropped from 0.74 ± 0.03 to 0.25 ± 0.04 (*p* = 0.025) and from 0.77 ± 0.04 to 0.50 ± 0.03 (*p* = 0.005) after 5 days of culture when 1 mM and 8 mM of the antioxidant were added to the 3D spheroidal droplet (Fig. 2A). But with the addition of 15 mM of NAC, no statistically significant difference was observed between day 1 (0.90 ± 0.02) and day 5 (0.86 ± 0.02) with *p* > 0.05.

To further calculate the number of viable to non-viable CMs present, the linear best-fit equations were used. % CV decreased from 82 ± 6% to 24 ± 3% (*p* = 0.0105) and from 83 ± 6% to 50 ± 2% (*p* = 0.025) after 5 days of culture when 1 mM and 8 mM of Tiron (Fig. 2A) were added respectively to the 3D spheroidal droplets. But with the addition of 15 mM of Tiron, no statistically significant difference was observed in % CV between day 1 (98 ± 5%) and day 5 (92 ± 6%) (ESI, Table 2A†) with *p* > 0.05.

With 1 mM and 8 mM of NAC (ESI, Table 2B†), the % CV dropped from 78 ± 3% to 25 ± 4% and from 82 ± 4% to 54 ± 3% respectively after 5 days of culture. But with the addition of 15 mM of NAC (ESI, Table 2B†), % CV remained relatively stable between day 1 (97 ± 2%) and day 5 (95 ± 2%) with *p* > 0.05. A similar trend was observed in CMs cultured in 2D plates with 1 mM, 8 mM, and 15 mM of Tiron (Fig. 2C and D). While OD trends and % CV were comparable between the 3D bioprinted spheroidal droplet scaffolds and CMs cultured in 2D cell cultures, 3D scaffolds potentially serve as a desirable microenvironment for CMs providing mechanical support and necessary biochemical cues for optimal cell, proliferation, and function.^41,42^ The actual number of live CMs derived from the best fit curve can be found in ESI, Fig. S8† (for 3D models) and ESI, Fig. S9† (for 2D models) respectively. All the trends were statistically significant between the varying time points studied.

### Confirmation of the cardioprotective effects of tiron and NAC in the presence of DOX

To further illustrate the cardioprotective effects of NAC and Tiron in the presence of DOX, live/dead images were acquired and quantified as depicted in Fig. 3A and B (3D models) and C and D (2D) respectively. To visualize such effects on cardiac cells, quantitative analysis was performed on the acquired images and results indicated that the % CV of CMs in the 3D bioprinted spheroidal droplets decreased from 85 ± 13% on day 1 to 42 ± 13% on day 5 (*p* = 0.015) (ESI, Table 3A†) when 1 mM Tiron was added, and from 89 ± 13% on day 1 to 52 ± 6% on day 5 (*p* = 0.025) when 8 mM of Tiron was added. But with the addition of 15 mM of Tiron, no statistically significant difference was observed in % CV between day 1 (95 ± 11%) and day 5 (93 ± 9%) with *p* > 0.05. With the addition of 1 mM of NAC (ESI, Table 3C†), % CV decreased from 84 ± 12% on day 1 to 52 ± 4% (*p* = 0.04) on day 5 and from 94 ± 11% on day 1 to 54 ± 3% (*p* = 0.002) on day 5 when 8 mM NAC was added. But with the addition of 15 mM of NAC, no statistically significant difference was observed in % CV between day 1 (93 ± 14%) and day 5 (96 ± 4%) with *p* > 0.05.

For the 2D control samples treated with NAC and Tiron (Fig. 3C and D), the % CV of cardiomyocytes decreased from 94 ± 7% on day 1 to 57 ± 9% on day 5 (*p* = 0.03) when 1 mM of Tiron (ESI, Table 3B†) was added and from 89 ± 13% on day 1 to 43 ± 9% on day 5 (*p* = 0.005) when 8 mM of Tiron was added. But with the addition of 15 mM of Tiron, no statistically significant difference was observed in % CV between day 1 (89 ± 22%) and day 5 (95 ± 6%) with *p* > 0.05. With 1 mM of NAC (ESI, Table 3D†), % CV decreased from 90 ± 7% on day 1 to 56 ± 8% (*p* = 0.015) on day 5 and from 90 ± 12% on day 1 to 49 ± 12% (*p* = 0.035) on day 5 when 8 mM NAC was added. But with the addition of 15 mM of NAC, no statistically significant difference was observed in % CV between day 1 (96 ± 5%) and day 5 (95 ± 6%) with *p* > 0.05. Quantitative analysis of representative live–dead images acquired when 3 mM, 5 mM, and 10 mM of NAC and Tiron were added can be found in ESI, Fig. S10 and S11† for 3D and 2D samples respectively. Images of negative control hydrogel scaffolds with DOX acquired using 43 DsRed filter can be found in ESI, Fig. S12.† % CV data collected from the live–dead assay further corroborates the cardioprotective role of Tiron and NAC against the induced cardiotoxic effects of doxorubicin as shown by other published research articles.^43,44^ The OD trends and % CV were comparable between the 3D bioprinted spheroidal droplet scaffolds and CMs cultured in 2D cell cultures validating the former. Because the 3D bioprinted spheroidal scaffolds serve as a desirable microenvironment for cells, in the future, other cell types can be seeded in the 3D bioprinted spheroidal scaffolds and utilized for drug screening and cytotoxicity testing.

### Mechanistic insights on DOX-induced cardiotoxicity

**Fig. 4:**
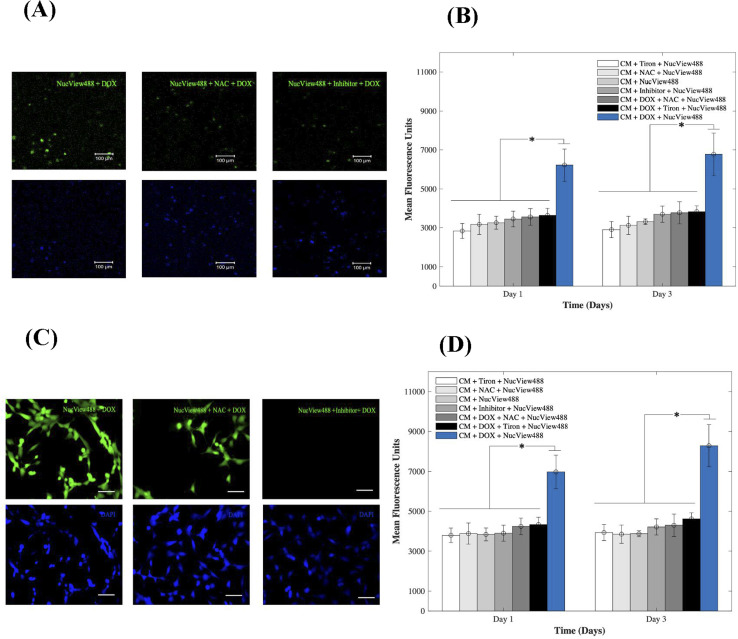
Evaluation of the caspase-3 activity of CMs. (A) In the 3D bioprinted spheroidal droplets representing fluorescence images of experimental and control groups of caspase-3 activated cardiomyocytes after adding 1 μm DOX and AOs compared to the control group captured on day 3 of culture. (B) Bar chart illustrating their relative mean fluorescence intensity. *p values were found to be all statistically different. The scale bar corresponds to 100 μm. (C) 2D samples representing fluorescence images of experimental and control groups of caspase-3 activated cardiomyocytes after adding 1 μm DOX and Tiron/NAC compared to the control group captured on day 3 of culture. (D) Bar chart illustrating their relative mean fluorescence intensity. *p values were found to be all statistically different. The scale bar corresponds to 80 μm.

To gain insight into the cardiotoxic effects induced by DOX on CMs and how those were mitigated by the addition of 15 mM of Tiron and NAC; Fig. 4, we examined caspase-3 activity in the 3D bioprinted spheroids (A, B) and in 2D samples (C, D). Experimental groups included CMs samples with DOX, AOs (Tiron & NAC), and NucView488 Cas-3 substrate (SUB), while control samples included CMs with caspase-3 inhibitor (Ac-DEVD-CHO), AOs (Tiron & NAC), and NucView488 Cas-3 substrate (SUB). Using a microplate reader, a significant difference (*p* = 0.001) was observed in the measured mean fluorescence units (MFU) expressing Cas-3 activity for the 3D bioprinted spheroidal droplets (Fig. 4A and B) between the group that contained DOX + SUB (6216 ± 823) and the groups that had Tiron + SUB (2829 ± 386); NAC + SUB (3172 ± 520); CM + SUB (3254 ± 335); inhibitor + SUB (3448 ± 404), DOX + NAC + SUB (3553 ± 430) and DOX + Tiron + SUB (3637 ± 362) at day 1 with *p* = 0.005. A similar trend in MFU was observed on day 3 but was not significant compared to day 1 (*p* > 0.05).

For 2D samples, a significant difference (*p* = 0.025) was observed in the MFU expressing cas-3 activity (Fig. 4C and D) between the group that contained DOX + SUB (6974 ± 841) and the groups that contained Tiron + SUB (3796 ± 363); NAC + SUB (3881 ± 531); CMs + SUB (3841 ± 321); inhibitor + SUB (3901 ± 399); DOX + NAC + SUB (4242 ± 417); and DOX + Tiron + SUB (4334 ± 371). A similar trend in MFU was observed on day 3 but was not significant compared to day 1 (*p* > 0.05). The NucView488 Cas-3 substrate, which was used to measure Cas-3 mediated apoptosis on CMs, was shown to have the highest emitted fluorescence in the group in which DOX was only added and the least fluorescence with Tiron/NAC or the Cas-3 inhibitor. This implied the initiation of the apoptosis pathway in CMs triggered by the caspase cell signalling pathway *via* DOX administration. But with the addition of strong antioxidants such as Tiron & NAC, cell survival was stimulated under such conditions and counteracted the effects of DOX.^45^ The thickness of hydrogel scaffolds can interfere with the passage of emitted fluorescent light, which is reflected in a slight but consistent decrease in average intensity among the experimental and control groups.^46^

### Confirmation of oxidative stress post-DOX addition

**Fig. 5:**
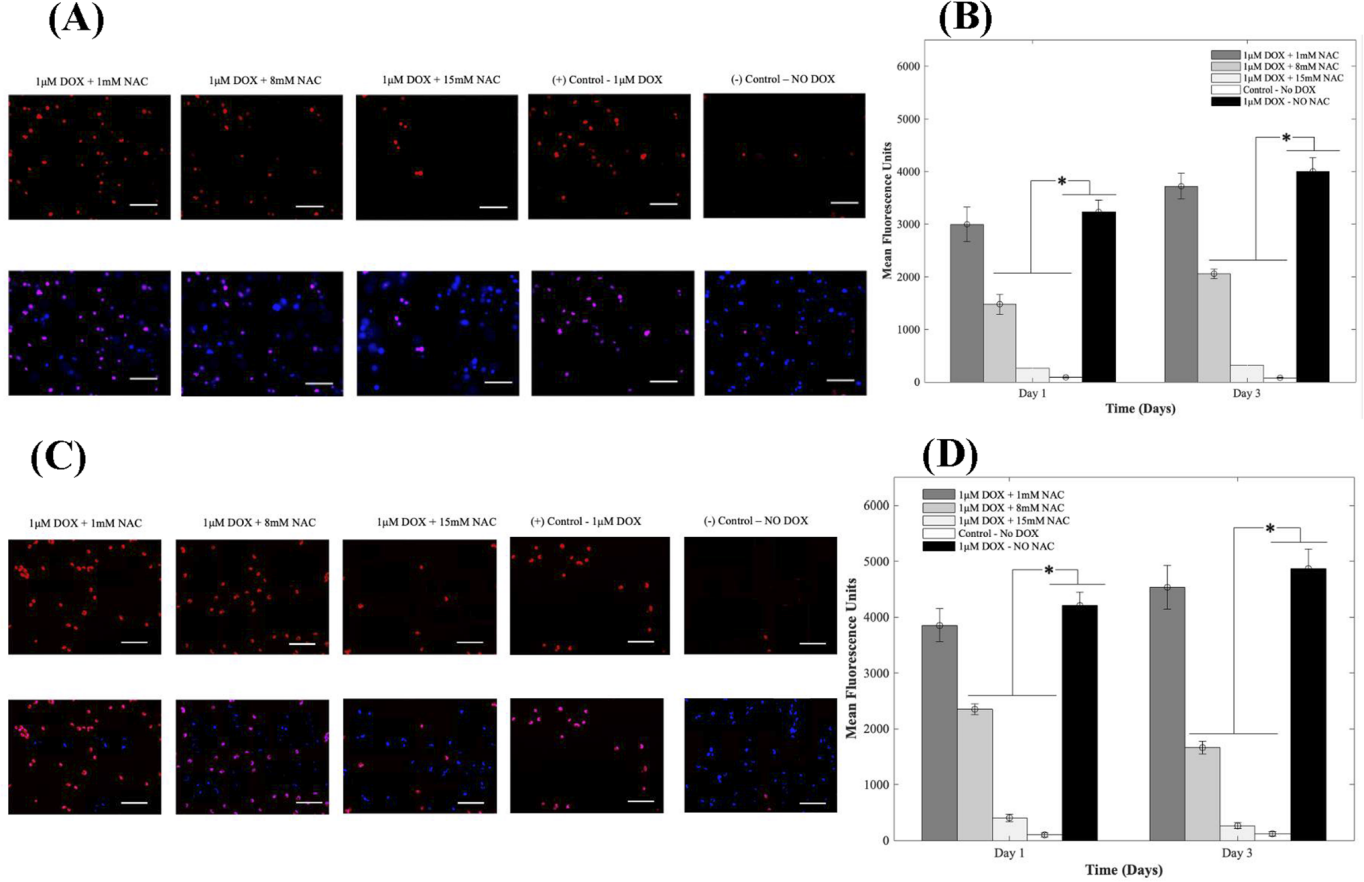
NAC antagonized doxorubicin-induced oxidative stress. Representative fluorescence images of experimental and control groups of CMs treated with 1, 8, and 15 mm of NAC and 1 μm DOX on day 3 of culture in 3D samples (A) and 2D samples (C). Bar chart illustrating intracellular ros production based on the relative mean fluorescence intensity (B). *p values were found to be all statistically different (*p* < 0.05). The scale bar corresponds to 100 μm in the 3D samples (B) and 2D samples (D).

Oxidative stress is a major player in DOX-induced cardiotoxicity. Moderate levels of ROS are vital for standard signal transduction processes, but elevated levels have been shown to be involved in various pathological conditions. Therefore, to study the effects of supplementing 1, 8, and 15 mM of NAC on CMs in the presence of 1 μM DOX, we examined DOX-induced oxidative stress using DHE. After oxidation, the superoxide indicator DHE binds with the cell's DNA, staining its nucleus a bright fluorescent red. Representative fluorescence images are shown in Fig. 5A and C respectively for 3D and 2D samples. From results depicted in A (3D samples) and C (2D samples), CMs exposed to DOX alone demonstrated a significant increase in fluorescence when compared to groups where NAC was supplemented. The increase in MFU is due to an increase in ROS production in samples where NAC was not supplemented and therefore indicated higher levels of DOX-induced oxidative stress. When compared to control samples (no DOX), quantitative analysis showed a 34-fold, 17-fold, and 3-fold increase at day 1 and a 48-fold, 27-fold and 4-fold in the MFU at day 3 of CMs in 3D bioprinted samples (Fig. 5B) with *p* = 0.002.

In 2D samples, quantitative analysis showed a 39-fold, 24-fold, and 4-fold increase on day 1 and a 39-fold, 14-fold and 2-fold in the mean fluorescent intensity on day 3 of CMs (Fig. 5D) when 1, 8, and 15 mM of NAC was added respectively. Hence, a reduction in MFU intensity was due to a decrease in ROS generation. The administration of AOs such as NAC significantly mitigated the DOX-induced oxidative stress compared to untreated groups. Data for intracellular ROS production when 1, 8, and 15 mM of Tiron were used can be found in ESI, Fig. S13.†

### Flow cytometric-based cell proliferation analysis on CMs

**Fig. 6:**
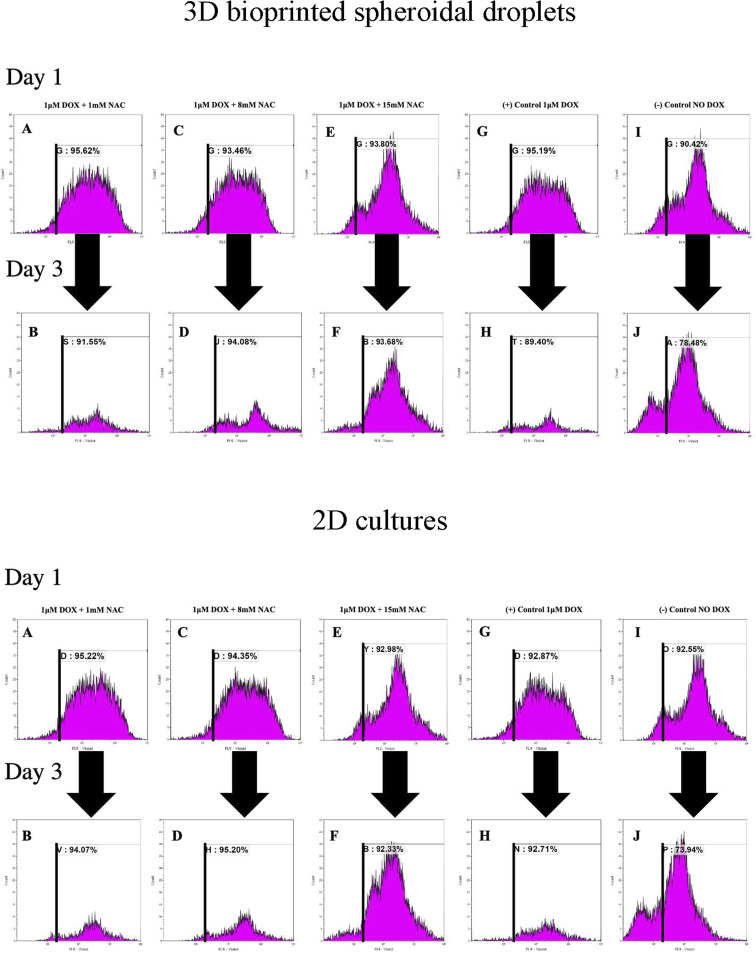
Analysis of the effect of DOX and NAC using FACS analysis within the 3D bioprinted spheroidal droplets and 2D cells culture samples. Cardiomyocytes were prestained with celltrace violet (CTV) and mixed with the bioink prior to 3d bioprinting. Cells were extracted from the scaffolds from the experimental and control groups and analyzed using a flow cytometer. Representative graphs (A, C, E, G, and I) indicate the %CTV+ after 1 day of culture and (B, D, F, H, and J) after 3 days of culture.

To analyse the proliferation trends of cardiomyocytes with DOX, CMs were prestained with CTV dye (ex405 nm/em450 nm) and extracted at each time point, and analysed based on the concept of dye dilution. This permitted us to study the trends of CMs proliferation seeded in the 3D bioprinted spheroidal droplets and in 2D samples (Fig. 6). While Tiron is a vitamin E analog and NAC is a non-toxic glutathione precursor, both are considered antioxidants that help protect cells from the damage caused by free radicals,^47,48^ therefore in this experiment only NAC-supplementation was studied. Experimental samples included CMs exposed to varying doses of only NAC (1, 8, and 15 mM; Fig. 6A–F for 3D samples/2D samples) and 1 μM DOX, whereas the positive control group (Fig. 6G–H for 3D samples/2D samples) included samples with DOX only (no NAC), and the negative control (Fig. 6I–J for 3D samples/2D samples) consisted of neither (no DOX, no NAC).

As shown in Fig. 6K, the average of %CTV+ of CMs at day 1 for 3D samples when 1 mM (91.87 ± 5.3%), 8 mM (88.07 ± 7.63%), 15 mM (87.75 ± 8.56%) of NAC was added respectively were not significant (*p* > 0.05) when compared to the positive (88.27 ± 9.79%) and negative (87.02 ± 4.82%) control group indicating a steady rate of proliferation of live CMs extracted from the 3D bioprinted spheroidal droplet at day 1.

On day 3, the average of %CTV+ of CMs at day 1 when 1 mM (88.84 ± 3.83%), 8 mM (93.11 ± 1.37%), 15 mM (88.16 ± 7.81%) of NAC and the positive control (87.29 ± 2.99%) were all statistically significant (*p* = 0.003) to the negative control (78.77 ± 0.4%) indicating that CMs did not significantly proliferate when 1 μM of DOX was added with varying concentration of NAC when compared to normal conditions. Moreover, the average change in %CTV + when 1 mM of NAC (−3.29 ± 0.02%), 8 mM NAC (5.72 ± 0.07%), 15 mM NAC (0.47 ± 0.008%), the positive control (−1.12 ± 0.01%) were all statistically significant (*p* = 0.015) to the negative control sample (−9.48 ± 0.05%).

For 2D samples, the average of %CTV+ (Fig. 6K) of CMs at day 1 when 1 mM (93.83 ± 1.97%), 8 mM (93.66 ± 0.98%), 15 mM (89.35 ± 5.14%) of NAC was added respectively were not significant (*p* > 0.05) when compared to the positive (92.64 ± 0.32%) and negative (89.22 ± 4.71%) control group indicating a steady rate of proliferation of live CMs at day 1.

On day 3, the average of %CTV+ of CMs at day 1 when 1 mM (94.19 ± 0.17%), 8 mM (94.04 ± 1.64%), 15 mM (93.72 ± 1.96%) of NAC and the positive control (94.21 ± 2.12%) were all statistically significant (*p* = 0.001) to the negative control (74.67 ± 1.03%) indicating that CMs did not significantly proliferate when 1 μM of DOX was added with varying concentration of NAC when compared to normal conditions. Moreover, the average change in %CTV + when 1 mM of NAC (0.38 ± 0.02%), 8 mM NAC (0.41 ± 0.007%), 15 mM NAC (4.89 ± 0.03%), the positive control (1.69 ± 0.02%) were all statistically significant (*p* = 0.025) to the negative control sample (−16.17 ± 0.04%). Shown in ESI, Table 4† are the average %CTV values expressed by cells in 3D and 2D platforms on days 1 and 3.

Results indicate that with 1 mM, 8 mM and 15 mM of NAC cells were not proliferating based on the concept of dye dilution between day 1 and day 3 compared to the negative control sample where DOX was not added. Positive controls included freshly isolated and prestained CMs with CTV while negative controls included freshly isolated and non-stained cells analysed using FACS (ESI, Fig. S14†).

### Gene expression and evaluation of CMs using qPCR

**Fig. 7:**
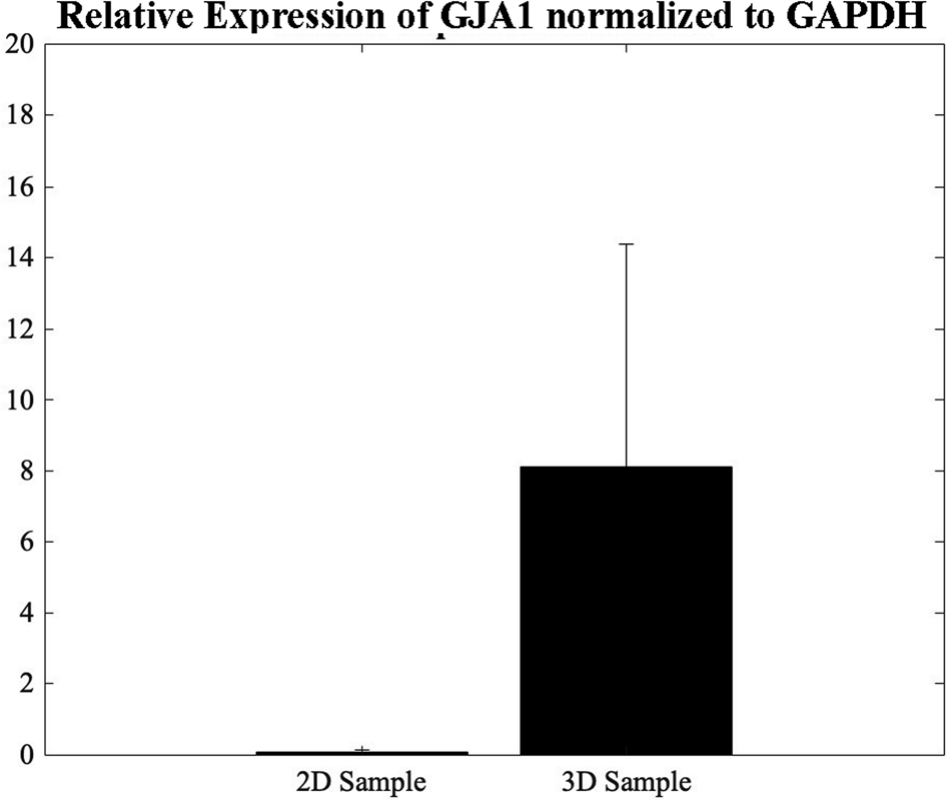
qPCR analysis. Relative expression levels of GJA1 in the 2D control samples and the 3D spheroidal droplets normalized to GAPDH.

The GJA1 gene delivers instructions for the transcription of a protein called connexion 43, one component of a large family of connexion proteins. Moreover, connexions play a major role in cell-to-cell communication by forming channels, or gap junctions.^49^ In an attempt to study the expression of GJA1, a gene from which CX-43 protein is translated, in the 3D bioprinted spheroidal droplets in comparison with 2D controls, CMs were extracted using both models on day 5 and analysed using a thermocycler. As shown in Fig. 7, results indicated statistical significance enhancement of the expression of the GJA1 gene between the 3D spheroidal droplet and the 2D control samples (*p* > 0.05).

## Discussion

Doxorubicin is a chemotherapeutic drug used to treat numerous diseases. However, patients experience its cardiotoxic effects limiting its use as an effective drug. Despite extensive research, the mechanisms by which doxorubicin kills cardiomyocytes have been elusive, and the exact mechanisms remain unknown.^50,51^

Doxorubicin-induced regulated cardiomyocyte death pathways include autophagy,^52^ ferroptosis,^43^ necroptosis,^52,53^ pyroptosis,^54^ and apoptosis.^52,55^ Autophagy is a homeostatic dynamic process by which cellular components, including organelles, are sequestered into membrane vesicles called autophagosomes which fuse with lysosomes for degradation under normal and stress conditions.^52^ Such conditions are caused by doxorubicin, and autophagy may be activated during doxorubicin treatment. While doxorubicin can stimulate autophagy, the deregulation of autophagy leads to uncontrolled cardiomyocyte death.^55^ Studies have shown that doxorubicin originally induces autophagy but then blocks it, resulting in the build-up of un-degraded autophagosomes. Therefore, the suppression of lysosomal proteolysis results in an accumulation of un-degraded vesicles, which leads to increased ROS production and CMs death.^48,52,55^ Another pathway that causes DOX-induced cardiotoxic effects is known as ferroptosis.^43^ It is characterized by the build-up of iron lipid peroxides, a significant source of ROS. In addition, DOX treatment increases the iron pool, especially in the mitochondria, which can be detrimental to cells. Doxorubicin also triggers another form of cell death known as necroptosis.^52,53^ While apoptosis and autophagy are considered “programmed cell death,” necrosis is regarded as “unprogrammed” due to deregulated activity involving the secretion of death-signalling cytokines. Pyroptosis is an inflammatory cell death and is widely recognized in the pathogenesis of cardiovascular diseases.^54^ It is accompanied by activating inflammasomes and caspase pathways, mainly caspase 3. The apoptotic pathway is the most studied programmed cell death pathway in DOX-induced cardiotoxicity. DOX treatment causes excess oxidative stress and mitochondrial damage triggering cell death pathways through the activation of caspase 9, which cleaves and activates caspase 3. DOX also activates apoptosis by other mechanisms, including upregulation of p53 resulting in extrinsic and intrinsic apoptosis.^52,55^ Doxorubicin has been well-characterized in lowering cell viability, possibly the most significant aspect of cardiotoxicity. The dose-dependent cardiotoxic effects of doxorubicin are well documented and revealed even at low cumulative doses.^50,51^ Patients prescribed DOX are at potential risk of its asymptomatic cardiotoxic side effects, such as elevated stress in the left ventricular wall leading to arrhythmias, heart failure to heart transplantation.^48,55^ The principal proposed mechanism of doxorubicin-induced cardiotoxicity is increased oxidative stress. The generation of ROS is the general route by which doxorubicin harms the myocardium. Furthermore, oxidative stress is associated with cardiomyocyte death, contributing to the doxorubicin-induced cardiotoxicity.^38,39^ Doxorubicin appears to cause damage to the mitochondria generating ROS and increasing superoxide formation by increasing endothelial nitric oxide synthase promoting intracellular hydroxide formation.^56,57^ Many studies have found various reasons behind DOX's cardiotoxicity, with a common factor whereby induces oxidative stress resulting in excessive ROS generation.^58^ Moreover, ROS production or oxidative stress promotes apoptosis and necrosis in cardiomyocytes developing severe cardiomyopathy.^59,60^ Oxidative stress from exposure to hydrogen peroxide (H_2_O_2_) and reactive oxygen species causes apoptosis in several cells and organ tissues, including cardiomyocytes.^61^ Cardiomyocytes exposed to DOX undergo apoptosis, and this effect is primarily attributed to the formation of oxygen free radicals and its intercalation into DNA and disruption of topoisomerase-ii-mediated DNA repair. Therefore, a treatment with various antioxidants has been proposed to mitigate cardiotoxicity caused by doxorubicin. NAC and Tiron have been identified as possible antioxidants effective at impeding apoptosis triggered by reactive oxygen species.^62^ While the ROS-scavenging role of NAC is evident, the process for their regulation of apoptosis is still ambiguous.^63^ The inhibition of apoptosis by antioxidants such as *N*-acetyl cysteine or vitamin E can further mitigate the outcomes of oxidative stress in the DOX-induced apoptosis.^64,65^ While cardiomyocytes are the target cell type of DOX-induced cardiotoxicity, we were particularly interested in assessing the activation of initiator caspases such as caspase-3 activity and the release of free radicals, which can cause oxidative damage to myocytes and lead to apoptosis and cell death.^38,59^ With maximum activity shown in the group with added doxorubicin, a lower activity was demonstrated in groups with NAC. In the present study, treatment of AC16 cardiomyocytes with antioxidants such as NAC and Tiron alleviated the DOX-induced oxidative stress in the 3D bioprinted spheroidal droplets and 2D samples by inhibiting the apoptotic pathway and decreasing the number of apoptotic cells.

## Conclusion

In this study, our key objective was to compare the traditional method for evaluating cytotoxicity using 2D tissue cultures with a developed 3D bioprinted spheroidal droplet model for high throughput testing. For this, we utilized a previously fabricated and optimized 3D bioprinted cardiac spheroidal model and evaluated the cardiotoxic effects of doxorubicin against AC16 cardiomyocytes. Unlike cell suspensions and tissue culture cellular monolayers, tissue-engineered constructs have a 3D structure to superiorly simulate the substantial impact that cell-to-cell and cell-to-matrix interactions influence cell behaviour in *in vivo* tissue and organ systems; a feature that 2D cell and tissue cultures cannot emulate well. In the future, the parameters of the spheroidal droplet model can be additionally optimized to express a wider range of human-derived tissue-engineered equivalents allowing the examination of various cells and their interactions in a more biomimetic environment. While 3D cell culture systems offer a better way of representing human tissue *in vitro*, the 3D bioprinted cardiac model can be utilized for other drug screening and drug cytotoxicity assays to evaluate how cells are affected by drugs, disease, or injury.

## Author contributions

Conceptualization, R. E. K, B. J.; formal analysis, R. E. K; investigation, R. E. K, C. L., S. R.; methodology, R. E. K, B. J.; project administration and supervision, B. J.; visualization, R. E. K.; writing original draft, R. E. K., C. L., B. J.; writing review and editing

## Conflicts of interest

There are no conflicts to declare.

## Supplementary Material

RA-013-D3RA00421J-s001
