# Supplementary material for: Demonstration of doxorubicin's cardiotoxicity and screening using a 3D bioprinted spheroidal droplet-based system
Source: RSC Adv. 2023 Mar 13;13(12):8338–51. doi: 10.1039/d3ra00421j (PMC10010162; doi:10.1039/d3ra00421j)
Supplement: RA-013-D3RA00421J-s001 [file RA-013-D3RA00421J-s001.pdf]

Electronic Supplementary Material (ESI) for RSC Advances.
This journal is © The Royal Society of Chemistry 2023

Supplementary Figures and Tables

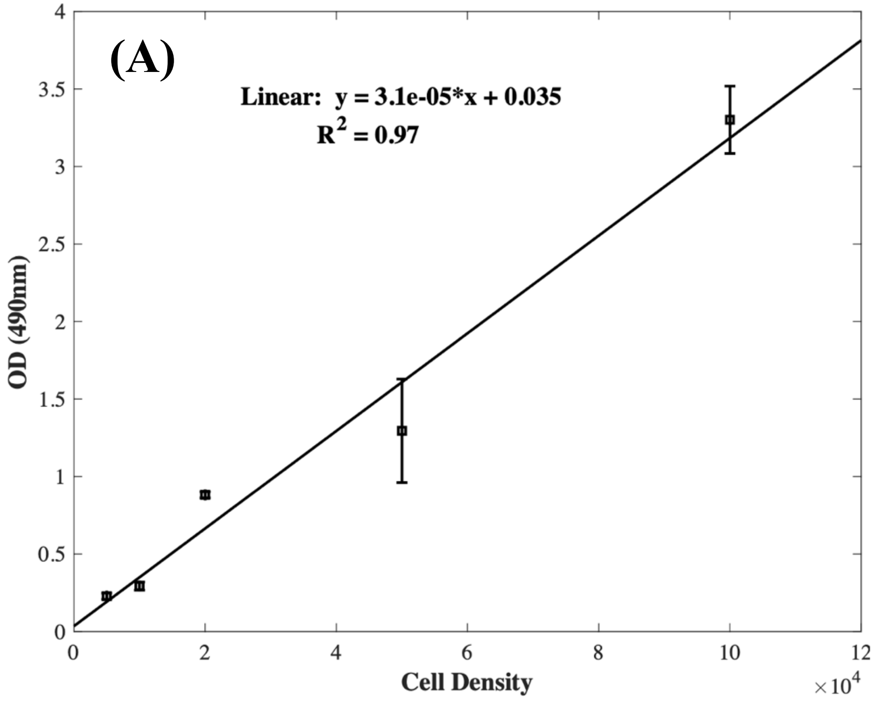

(B)	Day 1	Day 3	Day 5
	Number of LIVE cells	Number of LIVE cells	Number of LIVE cells
**0.4μM DOX**	27903 ± 620 cells	24677 ± 1234 cells	15645 ± 1203 cells
**0.6μM DOX**	26290 ± 1546 cells	20483 ± 1223 cells	12741 ± 889 cells
**0.8μM DOX**	23709 ± 1847 cells	9516 ± 1730 cells	2742 ± 457 cells
**1μM DOX**	21774 ± 920 cells	5322 ± 798 cells	62 ± 31 cells
**Control (NO DOX)**	28870 ± 931 cells	28548 ± 1241 cells	27903 ± 1240 cells

Figure S1. (A) Graph showing the linear best fit curve of the MTS assay data on CMs with various cell population densities in the 3D bioprinted spheroidal droplets. All values are expressed as Mean ± SEM of three replicates (n=3). (B) Table showing the amount of live CMs post-treatment with increasing concentrations of doxorubicin extrapolated from the linear best fit curve of MTS data during 5 days of culture.

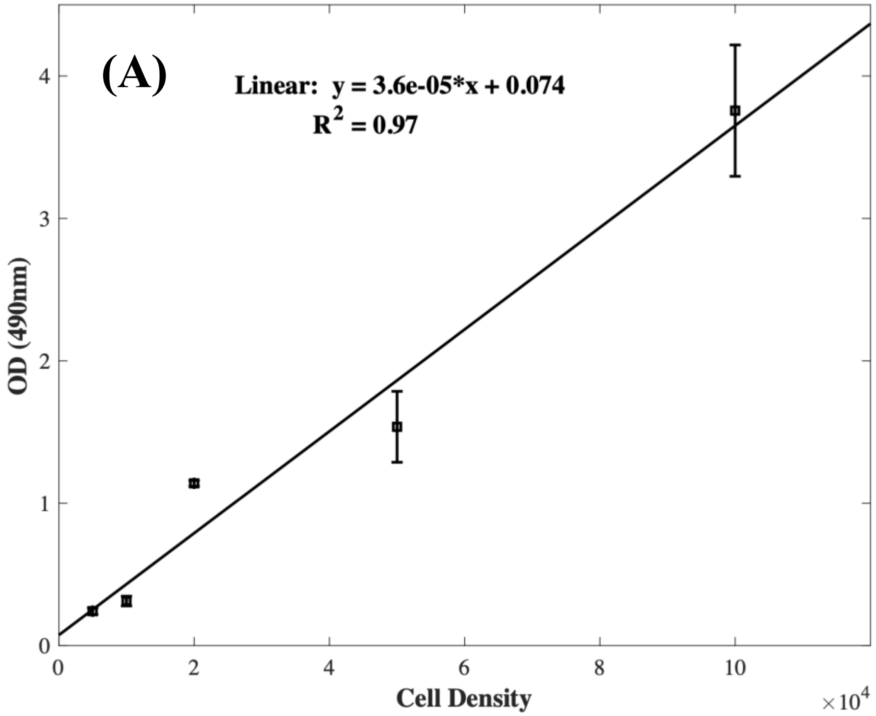

(B)	Day 1	Day 3	Day 5
	**Number of LIVE cells**	**Number of LIVE cells**	**Number of LIVE cells**
**0.4µM DOX**	26277 ± 1030 cells	21000 ± 1012 cells	17944 ± 997 cells
**0.6µM DOX**	25722 ± 1286 cells	16556 ± 988 cells	13500 ± 964 cells
**0.8µM DOX**	22944 ± 1529 cells	6278 ± 1256 cells	4056 ± 369 cells
**1µM DOX**	20444 ± 1262 cells	1833 ± 523 cells	284 ± 77 cells
**Control (NO DOX)**	27944 ± 1294 cells	27944 ± 776 cells	27944 ± 1294 cells

Figure S2. (A) Graph showing the linear best fit curve of the MTS assay data on CMs with various cell population densities in 2D samples. All values are expressed as Mean ± SEM of three replicates (n=3). (B) Table showing the amount of live CMs post-treatment with increasing concentrations of doxorubicin extrapolated from the linear best fit curve of MTS data during 5 days of culture.

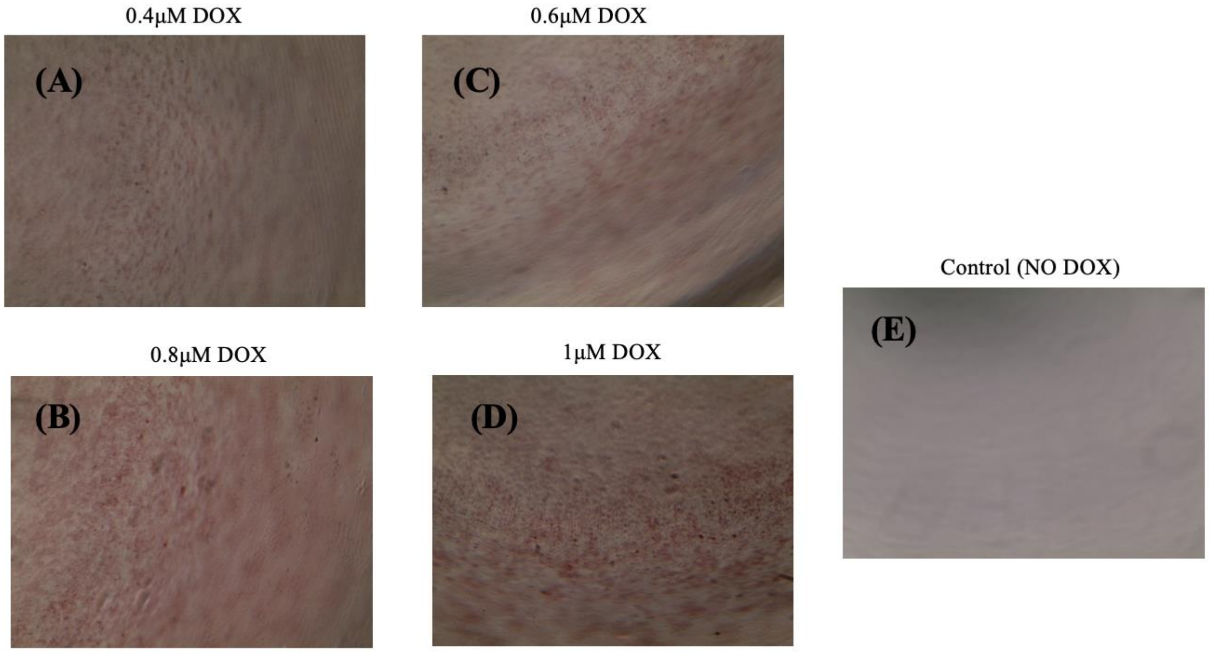

Figure S3: (A-D) Representative figures demonstrating the diffusion of DOX (0.4μM, 0.6μM, 0.8μM and 1μM) into the hydrogel scaffolds in comparison to the control sample scaffold (E) where DOX was not administered.

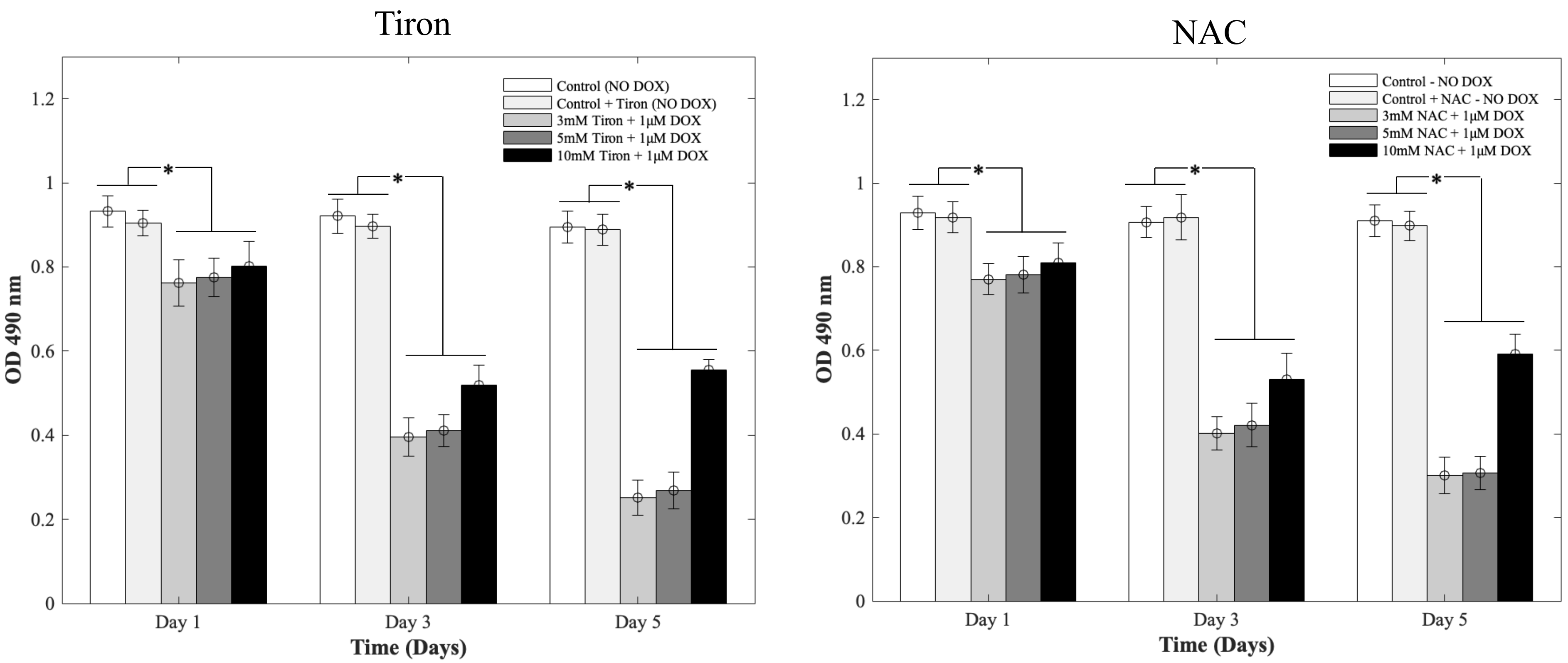

Figure S4. Optical density measurements for MTS assay of CMs in the 3D spheroidal droplets treated with increasing concentrations (3mM, 5mM, and 10mM) of (A) Tiron and (B) NAC respectively with 1μM DOX during 5 days of culture. *p values were found to be all statistically different.

(A)

Tiron	Day 1			Day 3			Day 5		
	OD Value	% Cell Viability (% CV)	Number of LIVE cells	OD Value	% Cell Viability (% CV)	Number of LIVE cells	OD Value	% Cell Viability (% CV)	Number of LIVE cells
3mM	0.76 ± 0.06	81 ± 6%	23387 ± 1846 cells	0.40 ± 0.05	41 ± 5%	11774 ± 1472 cells	0.25 ± 0.04	25 ± 4%	6935 ± 1110 cells
5mM	0.78 ± 0.05	83 ± 5%	24032 ± 1541 cells	0.41 ± 0.04	42 ± 4%	12096 ± 1180 cells	0.27 ± 0.04	27 ± 4%	7581 ± 1123 cells
10mM	0.80 ± 0.06	85 ± 6%	24677 ± 1851 cells	0.52 ± 0.05	55 ± 5%	15645 ± 1504 cells	0.56 ± 0.03	61 ± 3%	16935 ± 907 cells
Control (NO DOX)	0.93 ± 0.04	100%	28871 ± 1242 cells	0.92 ± 0.04	100%	28548 ± 1241 cells	0.90 ± 0.04	100%	27903 ± 1240 cells
Control + Tiron (NO DOX)	0.90 ± 0.03	100%	27903 ± 930 cells	0.90 ± 0.03	100%	27903 ± 930 cells	0.89 ± 0.04	100%	27581 ± 1239 cells

(B)

NAC	Day 1			Day 3			Day 5		
	OD Value	% Cell Viability (% CV)	Number of LIVE cells	OD Value	% Cell Viability (% CV)	Number of LIVE cells	OD Value	% Cell Viability (% CV)	Number of LIVE cells
3mM	0.77 ± 0.04	82 ± 4%	23710 ± 1232 cells	0.40 ± 0.04	42 ± 4%	11774 ± 1177 cells	0.30 ± 0.04	30 ± 4%	8548 ± 1140 cells
5mM	0.78 ± 0.04	84 ± 4%	24302 ± 1232 cells	0.42 ± 0.05	44 ± 5%	12419 ± 1478 cells	0.31 ± 0.04	31 ± 4%	8871 ± 1145 cells
10mM	0.81 ± 0.05	87 ± 5%	25000 ± 1543 cells	0.53 ± 0.06	57 ± 6%	15968 ± 1808 cells	0.59 ± 0.05	63 ± 5%	17903 ± 1517 cells
Control (NO DOX)	0.93 ± 0.04	100%	28870 ± 1242 cells	0.91 ± 0.04	100%	28226 ± 1241 cells	0.91 ± 0.04	100%	28226 ± 1241 cells
Control + Tiron (NO DOX)	0.92 ± 0.04	100%	28548 ± 1241 cells	0.92 ± 0.05	100%	28548 ± 1552 cells	0.90 ± 0.04	100%	27903 ± 1240 cells

Figure S5. Tables showing the percent cell viability of CMs in the 3D spheroidal droplets treated with the same concentrations of (A) Tiron and (B) NAC with 1μM DOX extrapolated from the linear best fit curve of MTS data.

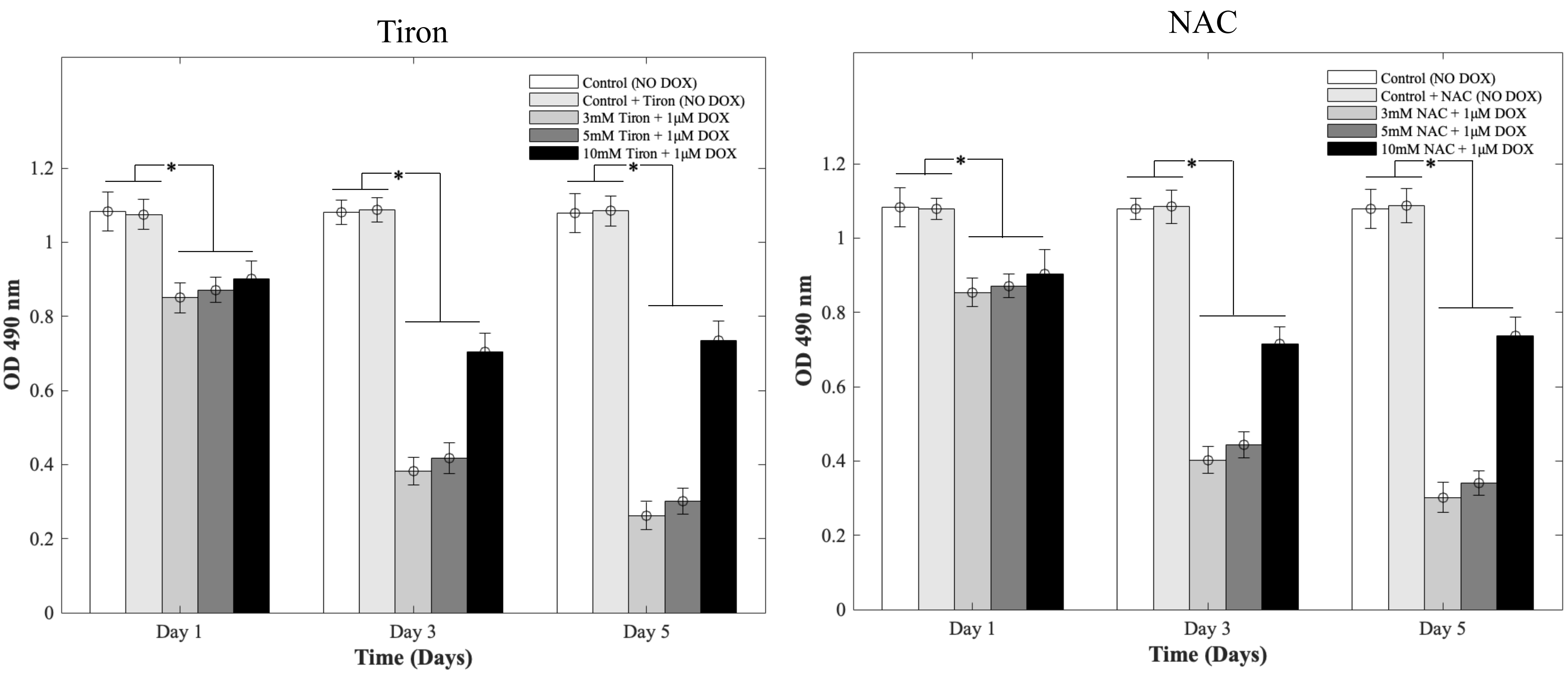

Figure S6. Optical density measurements for MTS assay of CMs grown on 2D samples with increasing concentrations (3mM,5mM, and 10mM) of (A) Tiron and (B) NAC with 1μM DOX after 5 days of culture. *p values were found to be all statistically different.

Tiron	(A) Day 1			Day 3			Day 5		
	OD Value	% Cell Viability (% CV)	Number of LIVE cells	OD Value	% Cell Viability (% CV)	Number of LIVE cells	OD Value	% Cell Viability (% CV)	Number of LIVE cells
**3mM**	0.85 ± 0.04	77 ± 4%	21556 ± 1014 cells	0.38 ± 0.04	30 ± 3%	8500 ± 895 cells	0.26 ± 0.04	18 ± 3%	5167 ± 795 cells
**5mM**	0.87 ± 0.03	79 ± 3%	22111 ± 762 cells	0.42 ± 0.04	34 ± 3%	9611 ± 915 cells	0.30 ± 0.04	22 ± 3%	6278 ± 837 cells
**10mM**	0.90 ± 0.05	82 ± 5%	22944 ± 1275 cells	0.71 ± 0.05	63 ± 4%	17667 ± 1244 cells	0.73 ± 0.05	65 ± 4%	18222 ± 1248 cells
**Control (NO DOX)**	1.08 ± 0.05	100%	27944 ± 1294 cells	1.08 ± 0.03	100%	27944 ± 776 cells	1.08 ± 0.05	100%	27944 ± 1294 cells
**Control + Tiron (NO DOX)**	1.08 ± 0.04	100%	27944 ± 1035 cells	1.09 ± 0.03	100%	28222 ± 777 cells	1.08 ± 0.04	100%	27944 ± 1035 cells

NAC	(B) Day 1			Day 3			Day 5		
	OD Value	% Cell Viability (% CV)	Number of LIVE cells	OD Value	% Cell Viability (% CV)	Number of LIVE cells	OD Value	% Cell Viability (% CV)	Number of LIVE cells
**3mM**	0.85 ± 0.04	77 ± 4%	21556 ± 1014 cells	0.40 ± 0.04	32 ± 3%	9056 ± 906 cells	0.30 ± 0.04	22 ± 3%	6278 ± 837 cells
**5mM**	0.87 ± 0.03	79 ± 3%	22111 ± 762 cells	0.44 ± 0.04	36 ± 3%	10166 ± 924 cells	0.34 ± 0.03	26 ± 2%	7389 ± 652 cells
**10mM**	0.90 ± 0.07	82 ± 6%	22944 ± 1785 cells	0.72 ± 0.05	64 ± 4%	17944 ± 1246 cells	0.74 ± 0.05	66 ± 4%	18500 ± 1250 cells
**Control (NO DOX)**	1.08 ± 0.05	100%	27944 ± 1294 cells	1.08 ± 0.03	100%	27944 ± 776 cells	1.08 ± 0.05	100%	27944 ± 1294 cells
**Control + NAC (NO DOX)**	1.08 ± 0.03	100%	27944 ± 776 cells	1.08 ± 0.05	100%	27944 ± 1294 cells	1.09 ± 0.05	100%	28222 ± 1295 cells

Figure S7. (A, B) Tables showing the percent % CV and number of live CMs grown on 2D samples extrapolated from the linear best fit curve of MTS data after 5 days of culture.

(A)		Day 1	Day 3	Day 5
		**Number of LIVE cells**	**Number of LIVE cells**	**Number of LIVE cells**
**Tiron**	**1mM**	23709 ± 1847 cells	11452 ± 881 cells	6613 ± 827 cells
	**8mM**	24032 ± 1849 cells	15000 ± 1200 cells	14032 ± 597 cells
	**15mM**	28225 ± 1551 cells	28226 ± 1241 cells	25645 ± 1545 cells
	**Control (NO DOX)**	28871 ± 1242 cells	28548 ± 1241 cells	27903 ± 1240 cells
	**Control + Tiron (NO DOX)**	28548 ± 1552 cells	28226 ± 931 cells	28225 ± 1241 cells

(B)		Day 1	Day 3	Day 5
		**Number of LIVE cells**	**Number of LIVE cells**	**Number of LIVE cells**
**NAC**	**1mM**	22741 ± 922 cells	12419 ± 1183 cells	6935 ± 1110 cells
	**8mM**	23710 ± 1232 cells	15645 ± 1203 cells	15000 ± 900 cells
	**15mM**	27903 ± 620 cells	26612 ± 1547 cells	26612 ± 619 cells
	**Control (NO DOX)**	28870 ± 1242 cells	28548 ± 1241 cells	27903 ± 1240 cells
	**Control + Tiron (NO DOX)**	28548 ± 931 cells	28548 ± 1552 cells	27903 ± 1240 cells

Figure S8. Tables showing the amount of live CMs in the 3D spheroidal droplets post-treatment with increasing concentrations of (A) Tiron and (B) NAC extrapolated from the linear best fit curve of MTS data during 5 days of culture.

(A)		Day 1	Day 3	Day 5
		**Number of LIVE cells**	**Number of LIVE cells**	**Number of LIVE cells**
**Tiron**	**1mM**	21278 ± 1013 cells	7944 ± 441 cells	3778 ± 900 cells
	**8mM**	21833 ± 1269 cells	13222 ± 1202 cells	12667 ± 717 cells
	**15mM**	26277 ± 773 cells	26833 ± 774 cells	26833 ± 774 cells
	**Control (NO DOX)**	27944 ± 1294 cells	27944 ± 776 cells	27944 ± 1294 cells
	**Control + Tiron (NO DOX)**	27944 ± 1035 cells	28222 ± 518 cells	28500 ± 78 cells

(B)		Day 1	Day 3	Day 5
		**Number of LIVE cells**	**Number of LIVE cells**	**Number of LIVE cells**
**NAC**	**1mM**	21556 ± 761 cells	9333 ± 911 cells	4611 ± 769 cells
	**8mM**	22388 ± 2035 cells	13222 ± 1202 cells	12667 ± 956 cells
	**15mM**	26833 ± 774 cells	26278 ± 1030 cells	26833 ± 774 cells
	**Control (NO DOX)**	27944 ± 1294 cells	27944 ± 776 cells	27944 ± 1294 cells
	**Control + Tiron (NO DOX)**	27944 ± 776 cells	27944 ± 1035 cells	28222 ± 1295 cells

Figure S9. Tables showing the amount of live CMs in 2D samples post-treatment with increasing concentrations of (A) Tiron and (B) NAC extrapolated from the linear best fit curve of MTS data during 5 days of culture.

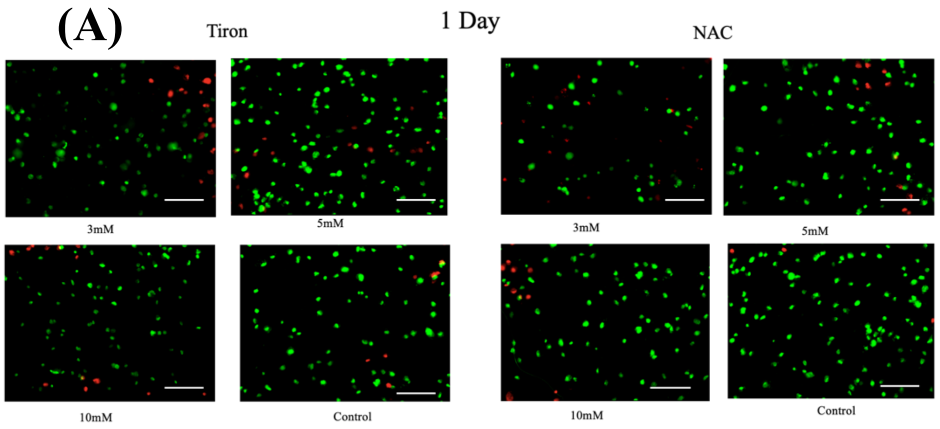

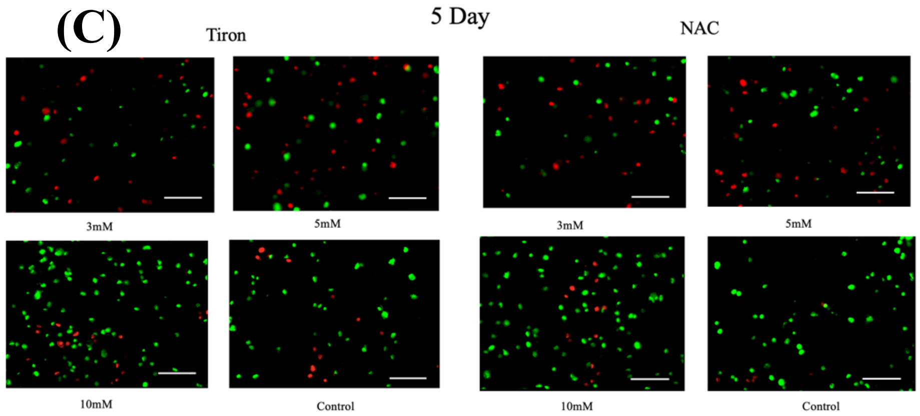

(B)

		Day 1<br>% Cell Viability<br>(% CV)	Day 5<br>% Cell Viability<br>(% CV)
**Tiron**	**3mM**	84 ± 21%	51 ± 24%
	**5mM**	87 ± 10%	50 ± 8%
	**10mM**	88 ± 8%	85 ± 12%
	**Control (NO DOX)**	94 ± 7%	80 ± 10%

(D)

		Day 1<br>% Cell Viability<br>(% CV)	Day 5<br>% Cell Viability<br>(% CV)
**NAC**	**3mM**	71 ± 6%	55 ± 3%
	**5mM**	90 ± 12%	54 ± 12%
	**10mM**	90 ± 13%	88 ± 5%
	**Control (NO DOX)**	98 ± 3%	95 ± 8%

Figure S10. Representative fluorescence images of live/dead staining of 3D bioprinted spheroidal scaffolds treated with increasing concentrations (3mM, 5mM, and 10mM) of Tiron and NAC and 1µM DOX. Live cells are stained in green by calcein AM and dead cells stained in red by ethidium homodimer after (A) 1 day and (C) 5 days of culture. (B, D) Tables representing the percent live/dead cells of CMs. The scale bar corresponds to 100µm.

(A)

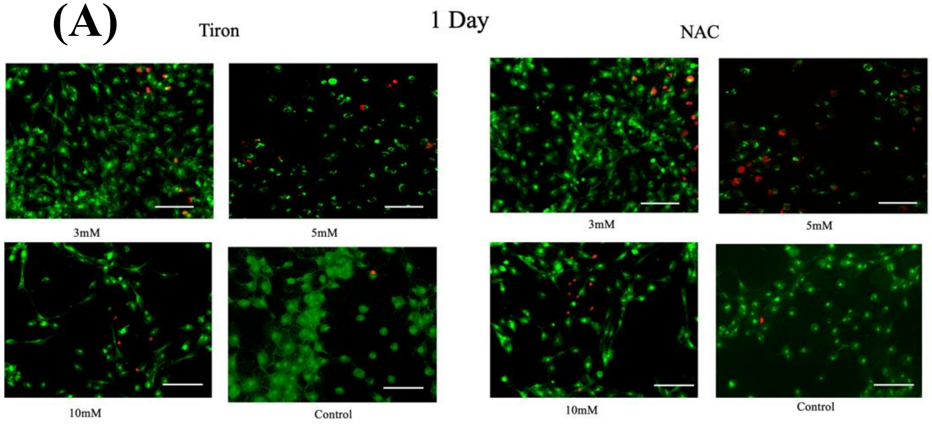

(C)

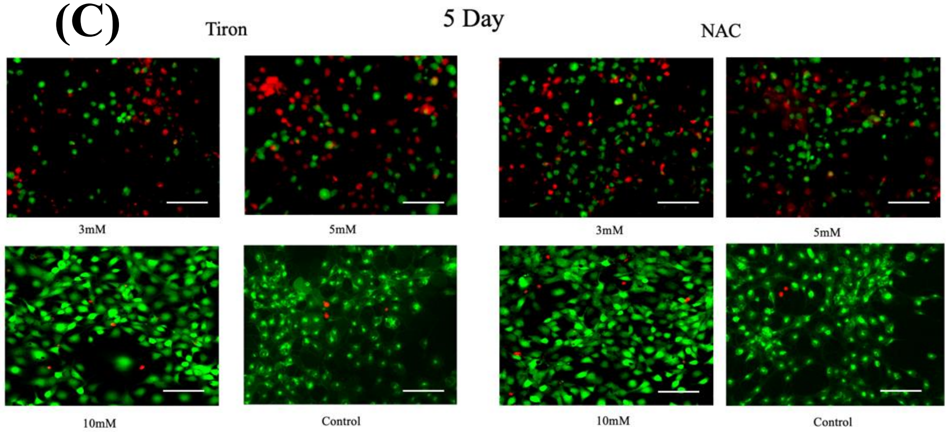

(B)		Day 1<br>% Cell Viability<br>(% CV)	Day 5<br>% Cell Viability<br>(% CV)
Tiron	3mM	94 ± 7%	45 ± 11%
	5mM	93 ± 6%	49 ± 9%
	10mM	92 ± 10%	93 ± 8%
	Control<br>(NO DOX)	99 ± 3%	97 ± 3%

(D)		Day 1<br>% Cell Viability<br>(% CV)	Day 5<br>% Cell Viability<br>(% CV)
NAC	3mM	94 ± 10%	57 ± 7%
	5mM	90 ± 8%	70 ± 14%
	10mM	92 ± 13%	90 ± 10%
	Control<br>(NO DOX)	99 ± 2%	99 ± 2%

Figure S11. Representative fluorescence images of live/dead staining of cardiomyocytes grown in 2D samples treated with increasing concentrations (3mM, 5mM, and 10mM) of Tiron and NAC and 1μM DOX. Live cells are stained in green by calcein AM and dead cells stained in red by ethidium homodimer treated with Tiron and NAC after (A) 1 day and (C) 5 days of culture. (B, D) Tables representing the percent live/dead cells of CMs. The scale bar corresponds to 100μm.

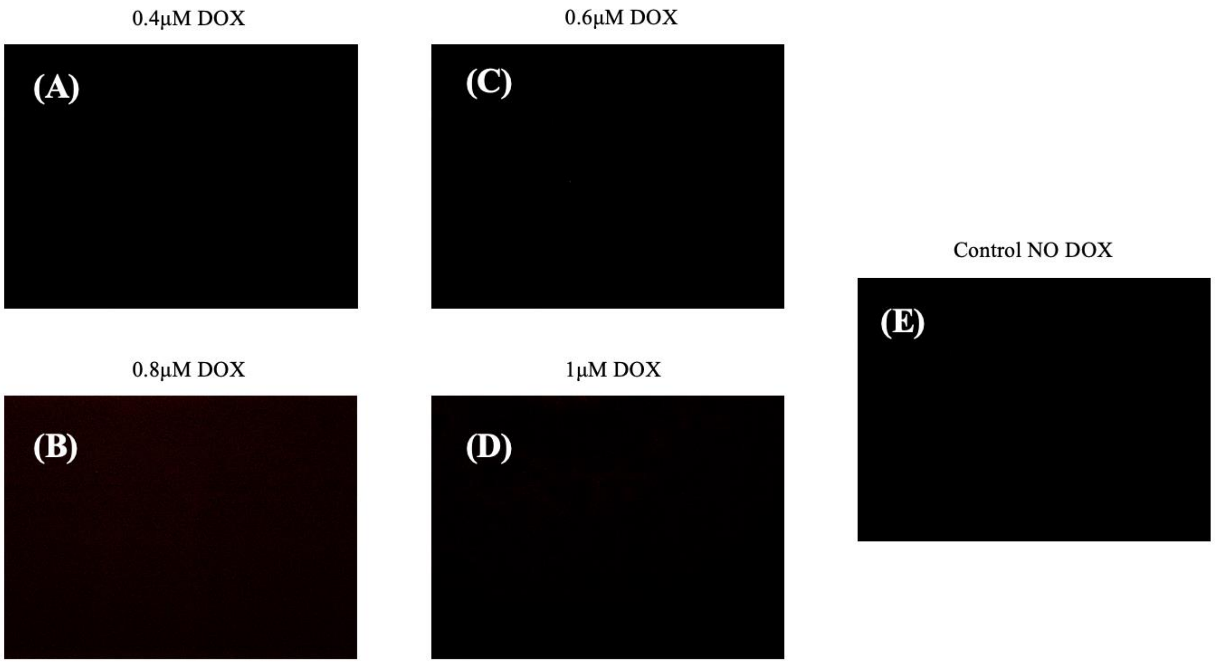

Figure S12: (A-D) Representative figures acquired using the 43 DsRed filter showing no fluorescence with different concentrations of DOX (0.4µM, 0.6µM, 0.8µM and 1µM) in comparison to the control sample scaffold (E) where DOX was not administered.

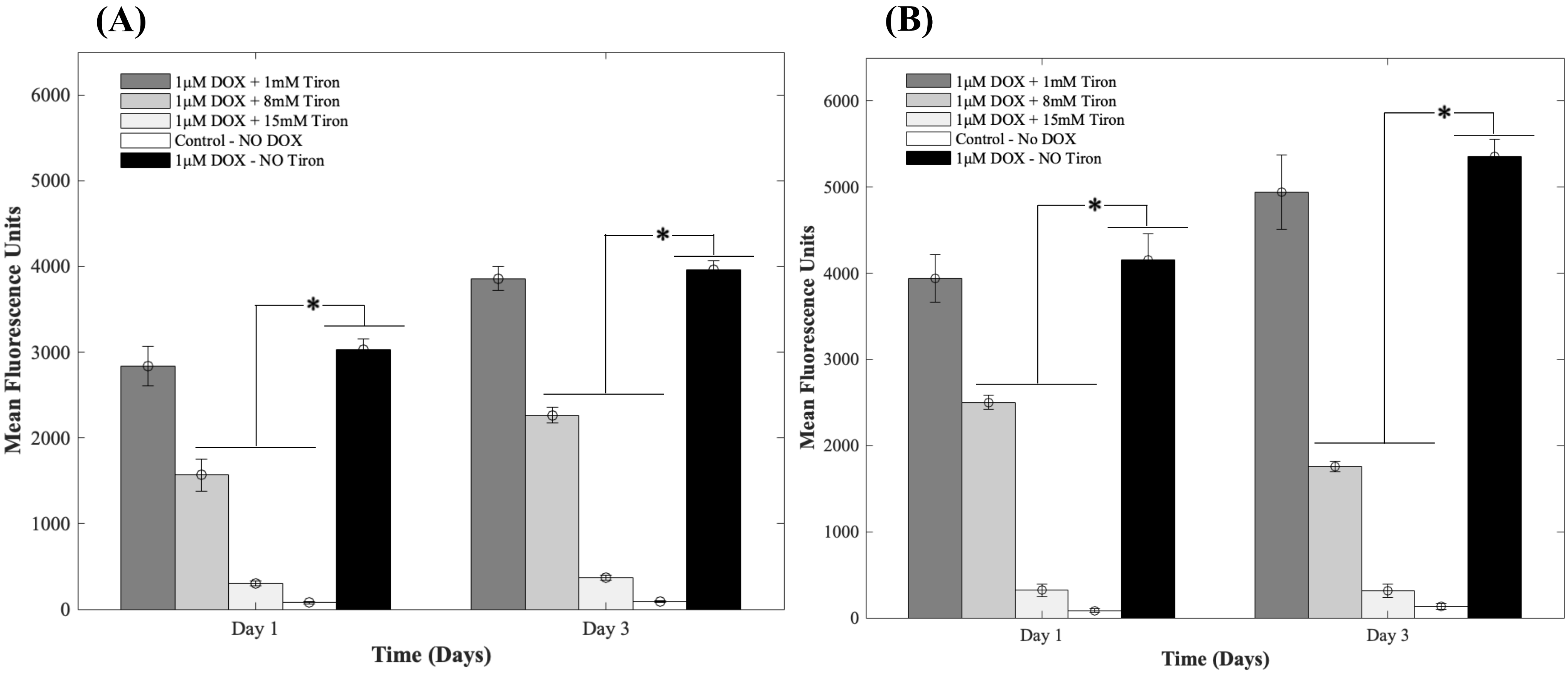

Figure S13. Bar charts illustrating intracellular ROS production using DHE staining of CMs treated with 1,8, and 15 mM of Tiron and 1μM DOX in (A) 3D spheroidal droplets and (B) 2D samples. **p* values were found to be all statistically different ($p<0.05$).

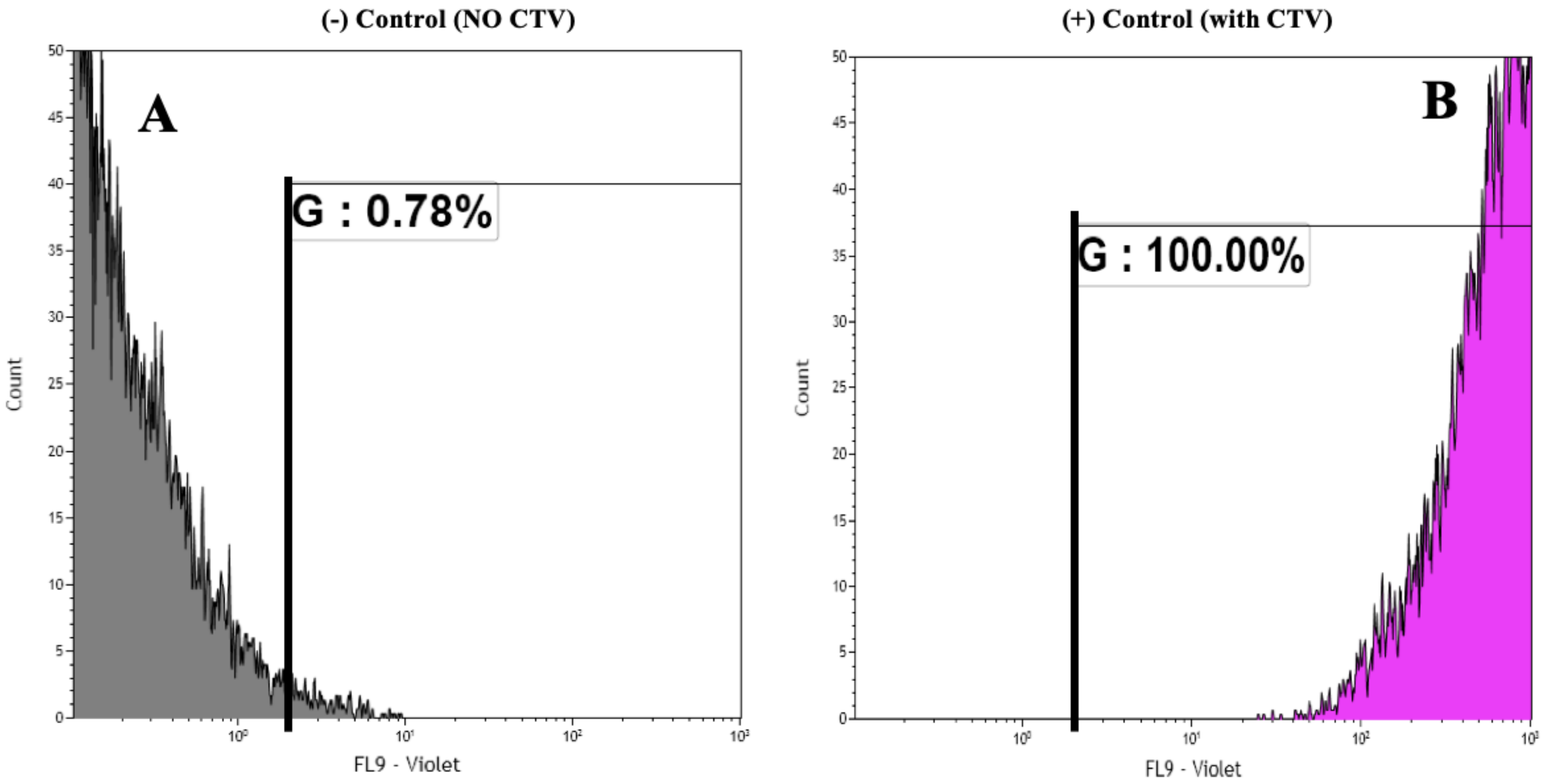

Figure S14. (A) Representative figure demonstrating a characteristic peak of a negative control samples (to the left). On the other hand, shown to the right is a characteristic peak of a positive control sample (B) whereby cells were prestained with CTV dye.

Supplementary Table 1. Dose responsive effects of DOX on CMs using 3D bioprinted spheroidal droplets (A) and 2D samples (B). Optical density measurements for MTS assay of CMs treated with increasing concentrations of DOX during 5 days of culture. Table showing the percent cell viability of CMs in 3D bioprinted spheroidal droplets (A) and 2D samples (B) treated with increasing concentrations of doxorubicin extrapolated from the linear best fit curve of MTS data (Figure S1-A and S8-A) over 5 days. The actual cell numbers used to generate % CV are shown in Figure S1-B and S8-B.

**(A)**	**Day 1**		**Day 3**		**Day 5**	
	**OD Value**	**% Cell Viability (% CV)**	**OD Value**	**% Cell Viability (% CV)**	**OD Value**	**% Cell Viability (% CV)**
**0.4μM DOX**	0.90 ± 0.02	97 ± 2%	0.80 ± 0.04	86 ± 4%	0.52 ± 0.04	56 ± 4%
**0.6μM DOX**	0.85 ± 0.05	91 ± 5%	0.67 ± 0.04	72 ± 4%	0.43 ± 0.03	46 ± 3%
**0.8μM DOX**	0.77 ± 0.06	82 ± 6%	0.33 ± 0.06	33 ± 6%	0.12 ± 0.02	10 ± 2%
**1μM DOX**	0.71 ± 0.03	75 ± 3%	0.20 ± 0.03	19 ± 3%	0.002 ± 0.001	0.22 ± 0.11%
**Control (NO DOX)**	0.93 ± 0.03	100%	0.92 ± 0.04	100%	0.90 ± 0.04	100%

**(B)**	**Day 1**		**Day 3**		**Day 5**	
	**OD Value**	**% Cell Viability (% CV)**	**OD Value**	**% Cell Viability (% CV)**	**OD Value**	**% Cell Viability (% CV)**
**0.4μM DOX**	1.02 ± 0.04	94 ± 4%	0.83 ± 0.04	75 ± 4%	0.72 ± 0.04	64 ± 4%
**0.6μM DOX**	1.00 ± 0.05	92 ± 5%	0.67 ± 0.04	59 ± 4%	0.56 ± 0.04	48 ± 3%
**0.8μM DOX**	0.90 ± 0.06	82 ± 6%	0.30 ± 0.06	22 ± 4%	0.22 ± 0.02	15 ± 1%
**1μM DOX**	0.81 ± 0.05	73 ± 5%	0.14 ± 0.04	7 ± 2%	0.011 ± 0.003	1.02 ± 0.28%
**Control (NO DOX)**	1.08 ± 0.05	100%	1.08 ± 0.03	100%	1.08 ± 0.05	100%

Supplementary Table 2. Quantitative analysis depicting the effects of supplementing Tiron/NAC on CMs using 3D spheroidal droplets (A, B) and 2D samples (C, D). Optical density measurements for MTS assay of CMs treated with increasing concentrations (1mM, 8mM, and 15mM) of Tiron and NAC with 1μM DOX. Tables representing the percent cell viability of CMs in 3D spheroidal droplets (A and B) and 2D samples (C and D) extrapolated from the linear best fit curve of MTS data during 5 days of culture. *p values were found to be all statistically different. The actual cell numbers used to generate % CV are shown in Figure S8.

Tiron

(A)

		Day 1		Day 3		Day 5	
		OD Value	% Cell Viability (% CV)	OD Value	% Cell Viability (% CV)	OD Value	% Cell Viability (% CV)
Tiron	1mM	0.77 ± 0.06	82 ± 6%	0.39 ± 0.03	40 ± 3%	0.24 ± 0.03	24 ± 3%
	8mM	0.78 ± 0.06	83 ± 6%	0.50 ± 0.04	53 ± 4%	0.47 ± 0.02	50 ± 2%
	15mM	0.91 ± 0.05	98 ± 5%	0.91 ± 0.04	99 ± 4%	0.83 ± 0.05	92 ± 6%
	Control (NO DOX)	0.93 ± 0.04	100%	0.92 ± 0.04	100%	0.90 ± 0.04	100%
	Control + Tiron (NO DOX)	0.92 ± 0.05	100%	0.91 ± 0.03	100%	0.91 ± 0.04	100%

NAC

(B)

		Day 1		Day 3		Day 5	
		OD Value	% Cell Viability (% CV)	OD Value	% Cell Viability (% CV)	OD Value	% Cell Viability (% CV)
NAC	1mM	0.74 ± 0.03	78 ± 3%	0.42 ± 0.04	44 ± 4%	0.25 ± 0.04	25 ± 4%
	8mM	0.77 ± 0.04	82 ± 4%	0.52 ± 0.04	55 ± 4%	0.50 ± 0.03	54 ± 3%
	15mM	0.90 ± 0.02	97 ± 2%	0.86 ± 0.05	93 ± 5%	0.86 ± 0.02	95 ± 2%
	Control (NO DOX)	0.93 ± 0.04	100%	0.92 ± 0.04	100%	0.90 ± 0.04	100%
	Control + NAC (NO DOX)	0.92 ± 0.03	100%	0.92 ± 0.05	100%	0.90 ± 0.04	100%

(C)

		Day 1		Day 3		Day 5	
		OD Value	% Cell Viability (% CV)	OD Value	% Cell Viability (% CV)	OD Value	% Cell Viability (% CV)
Tiron	1mM	0.84 ± 0.04	76 ± 4%	0.36 ± 0.02	28 ± 2%	0.21 ± 0.05	14 ± 3%
	8mM	0.86 ± 0.05	78 ± 5%	0.55 ± 0.05	47 ± 4%	0.53 ± 0.03	45 ± 3%
	15mM	1.02 ± 0.03	94 ± 3%	1.04 ± 0.03	96 ± 3%	1.04 ± 0.03	96 ± 3%
	Control (NO DOX)	1.08 ± 0.05	100%	1.08 ± 0.03	100%	1.08 ± 0.05	100%
	Control + Tiron (NO DOX)	1.08 ± 0.04	100%	1.09 ± 0.02	100%	1.10 ± 0.003	100%

(D)

		Day 1		Day 3		Day 5	
		OD Value	% Cell Viability (% CV)	OD Value	% Cell Viability (% CV)	OD Value	% Cell Viability (% CV)
NAC	1mM	0.85 ± 0.03	77 ± 3%	0.41 ± 0.04	33 ± 3%	0.24 ± 0.04	17 ± 3%
	8mM	0.88 ± 0.08	80 ± 7%	0.55 ± 0.05	47 ± 4%	0.53 ± 0.04	45 ± 3%
	15mM	1.04 ± 0.03	96 ± 3%	1.02 ± 0.04	94 ± 4%	1.04 ± 0.03	96 ± 3%
	Control (NO DOX)	1.08 ± 0.05	100%	1.08 ± 0.03	100%	1.08 ± 0.05	100%
	Control + NAC (NO DOX)	1.08 ± 0.03	100%	1.08 ± 0.04	100%	1.09 ± 0.05	100%

Supplementary Table 3. Live/Dead assay analysis representing the effects of supplementing Tiron/NAC on 3D spheroids droplets (A, C) and 2D samples (B, D) with CM. Tables representing the percent live/dead cells fluorescence staining of CMs grown on 3D spheroids droplet and 2D models treated with increasing concentrations of AO (1mM, 8mM, and 15mM) and 1μM DOX..

(A)

		Day 1 % Cell Viability (% CV)	Day 5 % Cell Viability (% CV)
Tiron	1mM	85 ± 13%	42 ± 13%
	8mM	89 ± 13%	52 ± 6%
	15mM	95 ± 11%	93 ± 9%
	Control (NO DOX)	94 ± 9%	94 ± 6%

(C)

		Day 1 % Cell Viability (% CV)	Day 5 % Cell Viability (% CV)
NAC	1mM	84 ± 12%	52 ± 4%
	8mM	94 ± 11%	54 ± 3%
	15mM	93 ± 14%	96 ± 4%
	Control (NO DOX)	95 ± 3%	94 ±12%

(B)

		Day 1 % Cell Viability (% CV)	Day 5 % Cell Viability (% CV)
Tiron	1mM	94 ± 7%	57 ± 9%
	8mM	89 ± 13%	43 ± 9%
	15mM	89 ± 22%	95 ± 6%
	Control (NO DOX)	95 ± 4%	94 ± 6%

(D)

		Day 1 % Cell Viability (% CV)	Day 5 % Cell Viability (% CV)
NAC	1mM	90 ± 7%	56 ± 8%
	8mM	90 ± 12%	49 ± 12%
	15mM	96 ± 5%	95 ± 6%
	Control (NO DOX)	93 ± 8%	94 ± 7%

Supplementary Table 4: Analysis of the effect of DOX and NAC using FACS analysis within the 3D bioprinted spheroidal droplets (A) and 2D (B). Cardiomyocytes were prestained with CellTrace Violet (CTV) and mixed with the bioink prior to the 3D bioprinting and 2D. Cells were extracted from the scaffolds and culture plates from the experimental and control groups and analyzed using a flow cytometer. Shown in A and B is the average of %CTV+ of prestained CMs on days 1 and 3.

(A)

		**1µM DOX + 1mM NAC**	**1µM DOX + 8mM NAC**	**1µM DOX + 15mM NAC**	**(+) Control 1µM DOX**	**(-) Control NO DOX**
**Day 1**	**Average %CTV+**	91.87±5.3%	88.07±7.63%	87.75±8.56%	88.27±9.79%	87.02±4.82%
**Day 3**	**Average %CTV+**	88.84±3.83%	93.11±1.37%	88.16±7.81%	87.29±2.99%	78.77±0.40
% Change (DAY 1 – DAY 3)		*-3.29±0.02%*	*5.72±0.07%*	*0.47±0.008%*	*-1.12±0.01%*	*-9.48±0.05%*

(B)

		**1µM DOX + 1mM NAC**	**1µM DOX + 8mM NAC**	**1µM DOX + 15mM NAC**	**(+) Control 1µM DOX**	**(-) Control NO DOX**
**Day 1**	**Average %CTV+**	93.83±1.97%	93.66±0.98%	89.35±5.14%	92.64±0.32%	89.22±4.71%
**Day 3**	**Average %CTV+**	94.19±0.17%	94.04±1.64%	93.72±1.96%	94.21±2.12%	74.67±1.03%
% Change (DAY 1 – DAY 3)		*0.38±0.02%*	*0.41±0.007%*	*4.89±0.03%*	*1.69±0.02%*	*-16.17±0.04%*
